# The Secret Life of the Anthrax Agent *Bacillus anthracis*: Bacteriophage-Mediated Ecological Adaptations

**DOI:** 10.1371/journal.pone.0006532

**Published:** 2009-08-12

**Authors:** Raymond Schuch, Vincent A. Fischetti

**Affiliations:** The Rockefeller University, New York, New York, United States of America; Cairo University, Egypt

## Abstract

Ecological and genetic factors that govern the occurrence and persistence of anthrax reservoirs in the environment are obscure. A central tenet, based on limited and often conflicting studies, has long held that growing or vegetative forms of *Bacillus anthracis* survive poorly outside the mammalian host and must sporulate to survive in the environment. Here, we present evidence of a more dynamic lifecycle, whereby interactions with bacterial viruses, or bacteriophages, elicit phenotypic alterations in *B. anthracis* and the emergence of infected derivatives, or lysogens, with dramatically altered survival capabilities. Using both laboratory and environmental *B. anthracis* strains, we show that lysogeny can block or promote sporulation depending on the phage, induce exopolysaccharide expression and biofilm formation, and enable the long-term colonization of both an artificial soil environment and the intestinal tract of the invertebrate redworm, *Eisenia fetida*. All of the *B. anthracis* lysogens existed in a pseudolysogenic-like state in both the soil and worm gut, shedding phages that could in turn infect non-lysogenic *B. anthracis* recipients and confer survival phenotypes in those environments. Finally, the mechanism behind several phenotypic changes was found to require phage-encoded bacterial sigma factors and the expression of at least one host-encoded protein predicted to be involved in the colonization of invertebrate intestines. The results here demonstrate that during its environmental phase, bacteriophages provide *B. anthracis* with alternatives to sporulation that involve the activation of soil-survival and endosymbiotic capabilities.

## Introduction

Anthrax is a rapidly lethal zoonotic disease caused by the bacterium *Bacillus anthracis*. Descriptions of anthrax as a scourge of livestock date back to the 1^st^ and 2^nd^ millennium B.C., and, because of its agricultural impact, the anthrax bacillus became the subject of seminal 19^th^ century microbiological studies into the cause and prevention of infectious disease by Robert Koch and Louis Pasteur [1]. Today, the spectre of anthrax endures as a biological weapon and a direct threat to human health [2]. Insightful research into areas ranging from population genetics and the molecular mechanisms of pathogenesis to therapeutics and diagnostics have dramatically broadened our knowledge of this pathogen [2], [3], [4], yet a major gap admittedly remains, concerning the environmental fate of *B. anthracis* following host death [1], [5], [6], [7].

**Figure 1:**
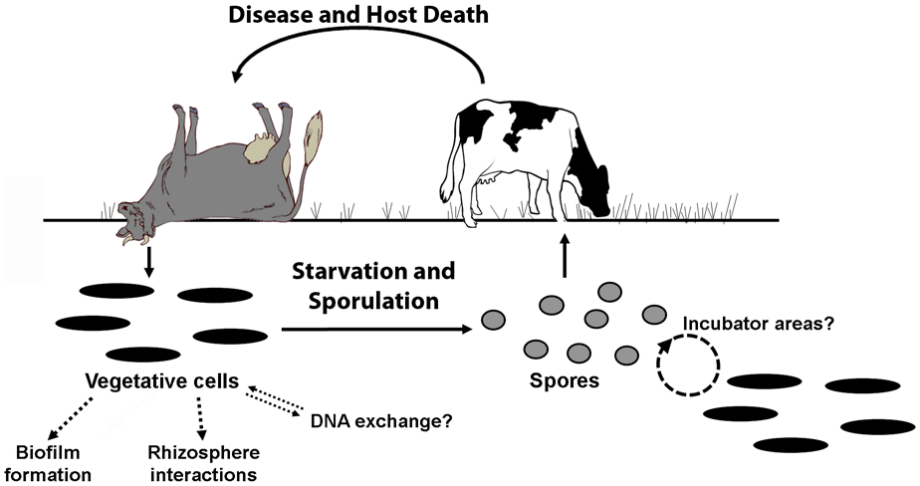
The *B. anthracis* lifecycle. Solid arrows trace a lifestyle in which dormant spores (the infectious cell-type) are ingested by grazing herbivores and then germinate to produce a vegetative cell-type that causes fulminant disease. After host death, processes like terminal hemorrhage and scavenger action release up to 10^9^ vegetative bacilli per milliliter of blood into the environment [1], [12]. While the fate of vegetative cells in the soil is unclear [1], [7], [27], [101], [102], the long-held model assumes that starvation and sporulation are the only option [8], [103]. Dashed arrows highlight some alternatives to sporulation implied in studies showing that *B. anthracis* forms biofilms (a preferred environmental state for soil bacteria) [25] and persists as a vegetative form in a model rhizosphere system [27]. The term “Incubator area” describes the hypothesis of Van Ness [24] that certain soil conditions may favor vegetative growth cycles prior to outbreaks. While it is unknown whether vegetative *B. anthracis* participates in DNA exchange in the soil, horizontal-gene-transfer is a driving force (in the face of selective pressure) for genetic variability and niche expansion in *B. cereus* and *B. thuringiensis* [6], [7], [28].

A central paradigm of the *B. anthracis* lifecycle described in figure 1 has the vegetative bacillus sporulating after host death and remaining dormant until encountering its next host [1], [8]. The ecological basis for this belief in a “spore-only” environmental fate is attributable to evidence like: 1) the durability and resistance properties of spores [9], including those of *B. anthracis* [10]; 2) laboratory demonstration of poor viability for some vegetative *B. anthracis* strains in water and soil microcosms [11], [12], [13]; and 3) surveys that show long-term spore contamination at anthrax carcasses [12], [14], [15], [16], [17], [18], [19], [20]. Nonetheless, the environmental fate of *B. anthracis* is considered enigmatic, owing largely to conflicting results from field studies. Several such studies, that include soil and water samplings at or near aging anthrax carcasses, show spores to be absent or only transient at enzootic areas and insufficient to support outbreaks [11], [12], [17], [21], [22], [23]. One study of the fate of *B. anthracis* at several anthrax carcasses for 60 months, shows that despite releases of up to 7×10^9^ vegetative cells, fewer than 5×10^4^ spores are ever detected [12]. Another confusing finding concerns long-term spore contaminations that are undiminished for years, despite exposure to conditions like wind, rain, and sunlight that should erode fixed reservoirs [1], [19]. Taken together, these results are hard to explain without environmental “incubator zones” originally proposed by Van Ness [24] in 1971 as sites of *B. anthracis* vegetative expansion prior to the sporulation necessary for outbreaks. In support of a vegetative phase, *B. anthracis* can grow as biofilms under *in vitro* static or laminar shear conditions [25], which is significant considering that biofilms are a preferred state for environmental organisms [26]. Saile and Koehler [27] have also shown that *B. anthracis* grows as a saprophyte in a model rhizosphere system. In light of these findings, it is important to note that *B. anthracis* shares a close genetic relationship with a group of highly successful soil organisms called the *B. cereus sensu lato* lineage [6], [28].

Taxonomically, *B. anthracis* forms three genetically homogeneous clades [29] in *B. cereus sensu lato*, a grouping that also includes *B. cereus sensu stricto* (referred to herein as *B. cereus*), a saprophyte, gut commensal, opportunistic pathogen and rhizosphere inhabitant, as well as the soil saprophyte *B. mycoides* and the insect pathogen *B. thuringiensis*. The pleomorphism within this lineage is astounding, considering that comparative genomics reveals relatively minor genetic differences; thus, a similar core set of genes supports various distinct lifestyles [6], [30], [31]. Indeed, the *B. anthracis* chromosome is largely distinguished only by four integrated, non-inducible prophages [32] and a single nonsense mutation that inactivates *plcR* [33]. PlcR is a transcriptional regulator of over 100 loci that enable *B. cereus* and *B. thuringiensis* to sense and respond to the environment [31], [34]. While these loci are encoded in *B. anthracis* [31], they are transcriptionally silenced by the *plcR* mutation. *B. anthracis* thus encodes the genetic capacity for environmental survival.

The anti-host functions of *B. anthracis* are actually encoded on the pXO1 and pXO2 virulence plasmids [8]. Large plasmids like pXO1 and pXO2 are quite common and highly variable within *B. cereus s.l*, and it is the genetic content of such plasmids and its interaction with core chromosomal loci that is an important driver of lifestyle diversity [28]. Examples of this in *B. anthracis* include the plasmid-encoded AtxA regulatory protein that may favor *plcR* inactivation [33], and plasmid-encoded signal sensor domains that can suppress the sporulation phenotype [35]. In this manner, *B. anthracis* virulence plasmids use regulatory proteins to control host gene expression and favor a pathogenic phenotype in an infected host.

In order to study environmental survival strategies, we considered contributions for important genetic elements other than plasmids in modulating *B. anthracis* phenotypes. Bacteriophages are such elements, with well described roles in transmitting genotypes that drive microbial diversity and niche expansion by stable infection or lysogeny [36]. Furthermore, phages in the lysogenic state, or prophages, can modify host phenotypes through several mechanisms, including the transmission of fitness genes that are not essential for the phage lifecycle and are called lysogen conversion factors. While such factors are traditionally described in terms of virulence proteins (e.g., toxins, adhesins, invasins, mitogenic factors, etc.) and the evolution of pathogenic phenotypes, they are now increasingly considered in promoting environmental functions for bacteria and include metabolic enzymes [37], [38] and transcriptional repressors for metabolic downshifts in nutrient-poor environments [39]. For *B. cereus s.l.*, the effects of lysogeny are not known despite numerous prophages [28], [40], [41] and a group of *B. cereus* and *B. thuringiensis* isolates that encode *B. anthracis*-infective phages [42], [43], [44]. Interestingly, these are among the most closely related isolates, genetically and/or phenotypically, to *B. anthracis* in *B. cereus s.l.* [28], [45]. As for the impact of lysogeny on *B. anthracis*, three major findings have been reported, including a *B. anthracis* phage conferring resistance to the soil antibiotic fosfomycin [46], a genotypically modified derivative of *B. anthracis* Sterne arising from phage infection [43], and a study from 1967 [47] suggesting that lysogeny is dispensable for virulence but may be required for efficient sporulation.

While no role has yet been assigned to bacteriophages in the *B. anthracis* lifecycle, a diverse set of inducible prophages has nonetheless been identified, even in well described laboratory strains like Sterne, Pasteur, and Vollum [43], [47], [48]. With environmental strains, it has been reported that *B. anthracis* soil isolates often contain phage-derived plaques upon subculture [27]. Furthermore, in studies of over 160 *B. anthracis* isolates from environments and diseased animals world-wide, >20% were stably infected with a variable array of inducible prophages, likely reflecting infections at different geographical locations [47], [48], [49], [50]. Free, infective phages for *B. anthracis* are also found in many environments, including sewage, tannery effluent, animal hair, soil and water at or near anthrax carcasses, as well as soil at non-endemic areas [18], [47], [48], [49], [50], [51], [52]. As a group, the phages of *B. anthracis* include members of all viral families, existing as 16–92 kb chromosomal and episomal prophages. Between the free environmental phages and the diversity of inducible prophages in *B. anthracis*, we reasoned that lysogeny impacts vegetative survival out of infected hosts in the environment.

Here, we report the first detailed analysis of lysogeny in *B. anthracis*. Using multiple distinct phages, we show that lysogeny profoundly alters the capacity of this organism to sporulate, produce exopolysaccharide, form biofilms, and survive long-term in the soil. We confirm, for the first time, the hypothesis of Louis Pasteur that *B. anthracis* colonizes earthworm intestines [53], and further show that this is dependent on lysogeny. A mechanism has been identified here, whereby phage-encoded sigma factors transcriptionally activate host-encoded loci that, in turn, induce novel phenotypes. These findings are consistent with a role for bacteriophages in ecological functioning and suggest that *B. anthracis* has a more dynamic environmental lifestyle than previously understood.

## Results

### Identification of environmental bacteriophages specific for *B. anthracis*

For this study, we used two distinct methods to collect eight novel bacteriophages that specifically infect *B. anthracis* (and not closely related *B. cereus* strains). For one method, we adapted a standard screening protocol to directly identify plaque-forming units (PFUs) or “free” phage particles within >150 different environmental extracts from an array of soil, fresh water and marine samples, as well as herbivore feces, rhizosphere and phylloplane washings, decaying organic matter, and soil invertebrate intestines. The *B. anthracis* laboratory strain ΔSterne was used as the susceptible host in these experiments because it lacks the inducible prophages that reside in many *B. anthracis* strains which could hamper our identification procedure. Ultimately, we identified PFUs from only three environments, including the gut of the earthworm *Eisenia fetida* (yielding Worm intestinal phages, Wip1 and Wip2), fern root systems (yielding the Fern rhizosphere phage, Frp1), and commercial potting soil (yielding the Soil lysogenic phage, Slp1). These phages were purified, amplified, and shown to infect *B. anthracis* ΔSterne and Sterne strains, but not the related *B. cereus* strain ATCC 14579 (Table 1). Partial genomic sequences were determined (and submitted to GenBank) for each phage to confirm their unique nature. M13 fingerprinting was also performed to distinguish each phage (data not shown).

**Table 1:** Growth of environmental phages on *B. anthracis* and *B. cereus* strains.

Phages	Source	*B. anthracis* ΔSterne	*B. anthracis* Sterne	*B. cereus* ATCC 14579
**Wβ**	Soil bacterium	2.1±0.3×10^8^	1.5±0.4×10^8^	<10
**Wip1**	Earthworm gut	1.3±0.5×10^8^	2.0±0.5×10^8^	<10
**Wip2**	Earthworm gut	6.7±1.2×10^8^	1.0±0.1×10^9^	<10
**Wip4**	Earthworm gut bacterium	4.5±0.5×10^5^	3.5±1.4×10^5^	<10
**Wip5**	Earthworm gut bacterium	4.8±0.6×10^7^	8.2±1.8×10^6^	<10
**Frp1**	Fern rhizosphere	6.9±1.4×10^7^	3.8±0.8×10^7^	<10
**Frp2**	Fern rhizosphere bacterium	5.4±1.0×10^8^	1.8±0.5×10^8^	<10
**Htp1**	Human tonsil bacterium	2.6±0.6×10^8^	1.3±0.2×10^9^	<10
**Slp1**	Potting soil	1.9±0.4×10^8^	2.1±0.5×10^8^	<10
**φ1615**	Soil bacterium	6.7±1.9×10^8^	9.5±0.1×10^8^	<10
**φ1047**	Earthworm gut bacterium	1.9±0.4×10^8^	2.1±0.5×10^8^	<10
**Bcp1**	Landfill soil	<10	2.1±0.5×10^8^	3.2±0.2×10^10^

Phages were isolated as free infective particles or inducible prophages from indicated environments. With the exception of Wβ, each phage was identified for this study. Maximum numbers of plaque forming units per ml of phage stock generated on each strain is indicated. Mean averages (± standard deviation) are shown for three to five experiments. “<10” indicates that plaque forming units were not detected.

A second method for bacteriophage identification focused on the inducible prophages of *B. anthracis*-like environmental organisms. Briefly, environmental samples were plated on non-selective BHI agar and the resulting colonies were ultimately screened for phenotypes and genotypes commonly associated with *B. anthracis* and *B. cereus* (Table S1). Notable *B. cereus s.l.* strains were then subjected to a prophage induction protocol, based on 24 hours of growth in BHI broth supplemented with fosfomycin, a soil antibiotic and inducer of lyosgenic phages from *B. cereus s.l.* [46]. Induced culture supernatants were then tested for PFUs on *B. anthracis* ΔSterne. In this manner, we identified Wip4 and Wip5 from earthworm gut bacteria, Frp2 from a fern rhizosphere bacterium and Htp1 from a human tonsil bacterium. As with the “free” phages identified above, the induced phages were found to be both genetically unique and specifically infective toward *B. anthracis* (Table 1).

### Characteristics of *B. anthracis* bacteriophages

**Figure 2:**
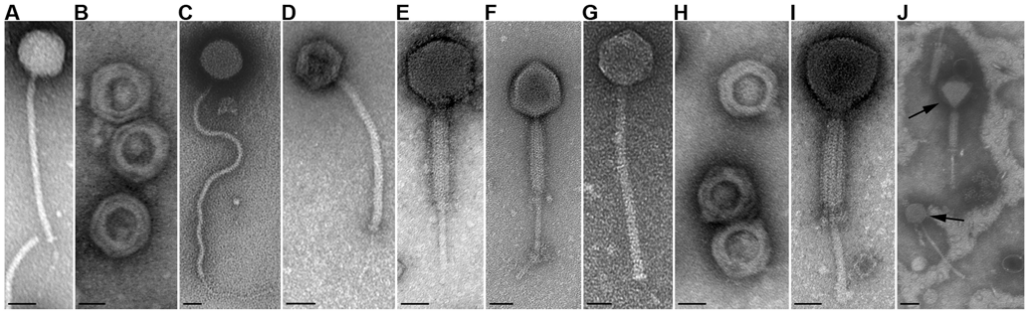
Transmission electron micrographs of bacteriophages negatively stained with 2% uranyl acetate. The bacteriophages infecting *B. anthracis* include, (A) Wβ, (B) Wip1, (C) Wip2, (D) Wip4, (E) Wip5, (F) Frp1, (G) Frp2, (H) Htp1, and (I) Bcp1. An extract from the gut of the earthworm *Eisenia fetida* is shown (J) with two distinct and uncharacterized phages indicated by arrows. Scale bars represent either 25 nm (A–I) or 50 nm (J).

The nature and variety of *B. anthracis* phages identified here were examined by electron microscopy. For comparison, we included the well described lysogenic *B. anthracis* bacteriophage, Wβ [46]. Ultimately, we observed three distinct morphotypes representing three viral families. The Wip2, Wip4, and Frp2 phages (Figure 2C, 2D and 2G) were most similar to Wβ (Figure 2A); their icosahedral heads and long, flexible, non-contractile tails are hallmarks of the *Siphoviridae* family of bacteriophages. Here, Wip2 is notable, not for an average head size of 60±1.5 nm (mean±SD, *n* = 50), but for a long tail length of 420±36 nm (*n* = 25) when compared to the average *Siphoviridae* length of 150 nm [54]. The Wip5 and Frp1 phages (Figure 2E and 2F) form a distinct group classified as *Myoviridae*, based on their icosahedral heads and short, thick, contractile tails. Wip1 and Htp1 are tail-less phages (Figure 2B and 2H) with isometric heads and are *Tectiviridae*; as with such phages, both Wip1 and Htp1 produced tail-like tubes after adsorption or chloroform treatment (data not shown). Our findings indicate that each particular worm gut has multiple distinct phage types as illustrated in Figure 2J, with a micrograph of bacteria-free (sterile-filtered) gut fluid containing undefined extracellular phages.

### The impact of lysogeny on colony morphology

To discern a role for environmental phages in the *B. anthracis* lifecycle, we infected ΔSterne with several phages from Table 1 and evaluated phenotypes of the resulting lysogens. All of the phages formed turbid plaques and were thus expected to be temperate phages capable of lysogeny. Indeed, lysogens recovered from plaques obtained with each phage were quite stable. In an analysis of spontaneous prophage curing, the loss of each phage was observed at a frequency ≤10^−4^ (Table S2), which is similar to that observed for stable λ phage lysogens [55]. The presence of putative phage integrase- and recombinase-encoding loci in the genomic sequences of several phages in this study suggests that lysogeny in most cases is associated with phage integration.

The colony morphology of ΔSterne lysogens was varied, based on the lysogenic phage. After a 24 hour incubation at 30°C, the Wip4, Wip5, Frp1, and Htp1 lysogens all presented flat, large, and opaque colonies that were quite distinct from a smaller, white, and matte morphology seen here with ΔSterne alone and the Wip1, Wip2, Frp2, Slp1, and Wβ lysogens. We confirmed this difference by re-generating all the lysogens using a liquid culture infection method. Here, lysogeny was readily detected and again the Wip4, Wip5, Frp1 and Htp1 lysogens all formed large and opaque colonies (examples seen in Figure S1A and S1B).

An interesting exception among the phages in this study concerns Bcp1 (for *B*. *cereus* phage), a member of the *Myoviridae* family (Figure 2I) that forms plaques on *B. cereus* and *B. anthracis* Sterne, but not *B. anthracis* ΔSterne (Table 1). Bcp1 nonetheless adsorbed to *B. anthracis* ΔSterne (Figure S2A and S2B, Table S3) and eventually yielded lysogens. The ΔSterne/Bcp1 lysogens were, however, only found in biofilm-like adherences at the liquid-air interface of 3 month infection cultures. It is likely that over long-term incubation, rare phage-sensitive derivates became stably infected and formed the observed biofilms. Non-lysogens were not observed in these structures. After subculture to BHI agar, the ΔSterne/Bcp1 lysogen also produced the large-colony phenotype (distinct from ΔSterne).

### The impact of lysogeny on sporulation

Since the distinctly larger colonial phenotype of Wip4, Wip5, Frp1, Htp1 and Bcp1 lysogens after 24 hours of growth on agar medium could be attributable to a faster growth rate, we followed growth in liquid M9 minimal medium. Indeed, each of these lysogens doubled in 37–39 minutes while ΔSterne and the Wip1, Wip2, Frp2, Slp1, and Wβ lysogens doubled in 61–64 minutes. Fast growing *B. anthracis* derivatives that form large and opaque colonies also appear after long-term storage [56] or growth at high temperature [47] and correspond to non-sporulating or asporogenous (Spo^−^) forms. To determine whether the ΔSterne lysogens described here were also Spo^−^, we examined sporulation in 24 hour Leighton-Doi (LD) cultures grown with aeration at 37°C. As expected, the large-colony Wip4, Wip5, Frp1, Htp1, and Bcp1 lysogens did not sporulate in conditions that yielded >10^8^ spores ml^−1^ for ΔSterne and the small-colony Wip1, Wip2, Frp2, Slp1, and Wβ lysogens (Table 2). Interestingly, when these Spo^−^ lysogens were manipulated to lose (or cure) their respective prophages, the resulting phage-free strains reverted to Spo^+^ (Table 2). These findings indicate that certain *B. anthracis* prophages can act to block sporulation.

**Table 2:** Formation of mature spores by *B. anthracis* ΔSterne and its lysogens under varying temperature and aeration conditions.

Strain (+ lysogenic phage)	37°C, good aeration	24°C, poor aeration
**ΔSterne**	4.5±1.0×10^8^	<10
** +Wβ**	6.9±0.4×10^8^	4.9±1.0×10^5^
** +Wip1**	6.5±0.2×10^8^	9.3±1.6×10^5^
** +Wip2**	5.0±0.4×10^8^	1.0±1.0×10^6^
** +Wip4**	<10	<10
** +Wip5**	<10	<10
** +Frp1**	<10	<10
** +Frp2**	5.4±0.5×10^8^	8.6±1.1×10^5^
** +Slp1**	5.1±0.7×10^8^	8.1±1.0×10^5^
** +Htp1**	<10	<10
** +Bcp1**	<10	<10
**ΔSterne (Wip1 cured)**	3.1±1.5×10^8^	<10
**ΔSterne (Wip2 cured)**	3.5±0.4×10^8^	<10
**ΔSterne (Wip4 cured)**	2.6±1.0×10^8^	<10
**ΔSterne (Wip5 cured)**	3.5±1.3×10^8^	<10
**ΔSterne (Frp1 cured)**	3.1±1.1×10^8^	<10
**ΔSterne (Frp2 cured)**	4.0±1.0×10^8^	<10
**ΔSterne (Slp1 cured)**	1.0±1.0×10^8^	<10
**ΔSterne (Bcp1 cured)**	2.5±1.0×10^8^	<10
**ΔSterne/pASD2**	5.2±0.5×10^8^*	<10
**+P-** ***bcp25,26*** ** (+353 bp promoter)**	<10*	<10
**+P-** ***wip38,39*** ** (+258 bp promoter)**	<10*	<10
** +** ***bcp25,26*** ** (promoterless)**	6.9±0.1×10^6^*	<10
** +** ***wip38,39*** ** (promoterless)**	7.3±0.9×10^6^*	<10
**ΔSterne+Bcp1/pASD2::** ***bcp25***	2.9±1.0×10^5^	<10
**ΔSterne+Wip4/pASD2::** ***wip39***	8.0±1.0×10^6^	<10

Cultures of *B. anthracis* ΔSterne (+ lysogenizing phage) and phage-cured derivatives were incubated in LD sporulation medium either 1 day at 37°C with aeration at 150 rpm or 5 days at 24°C with aeration at 75 rpm. The number of mature, heat-resistant spores per ml of culture was determined and is shown. Values are mean averages (± standard deviation) from three to five experiments. “^*^” denotes the fact that experiments were performed at 30°C (rather than 37°C) to maintain the self-replicating form of pASD2.

This ability of infecting phages to induce the Spo^−^ phenotype was further examined. Infections of ΔSterne with Wip4 were performed over a range of MOIs and the resulting colonies were analyzed for both lysogeny and the Spo^−^ phenotype. All resulting lysogens were Spo^−^ and they were pronounced even at the lowest MOI (Figure S1C). Furthermore, only live and infective phages (not heat-inactivated forms) resulted in Spo^−^ colonies, suggesting that lysogeny and not a physical interaction with the bacterial surface is needed. We next performed a more detailed comparison of lysogens obtained immediately after infection with the “Spo^+^” (Wβ and Wip2) and “Spo^−^”phages (Wip4, Wip5, and Frp1). While lysogeny was readily detected for both classes, the Wβ and Wip2 lysogens were invariably Spo^+^ and the Wip4, Wip5, and Frp1 lysogens were invariably Spo^−^ (Table S4). These results, taken with findings that phage-curing restores the Spo^+^ phenotype, suggest that lysogeny drives the asporogenous phenotype.

**Figure 3:**
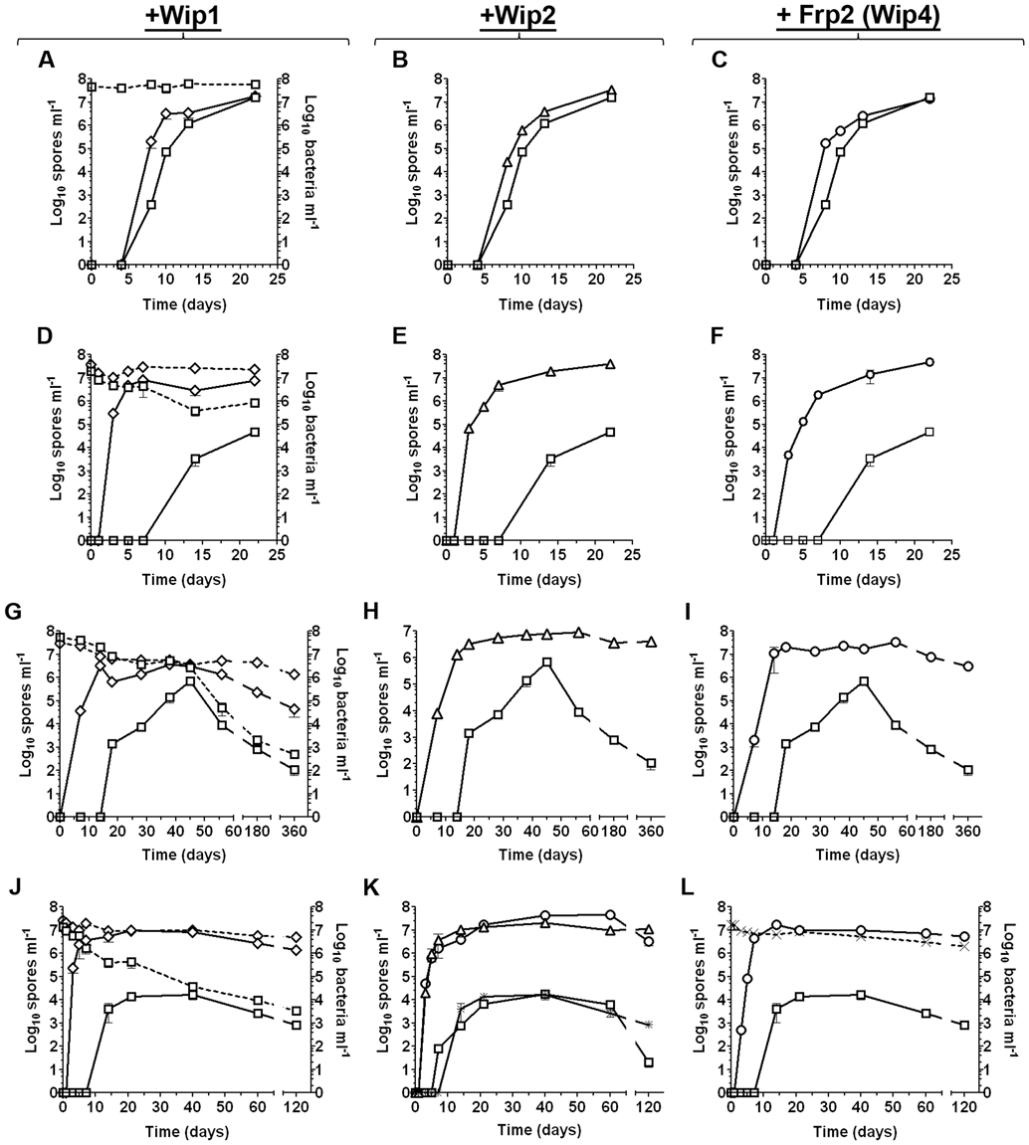
Certain lysogens of *B. anthracis* sporulate rapidly. Total bacterial cells (vegetative cells and spores; dotted lines) and spores (solid lines) in liquid sporulation cultures were determined over time. Values are mean averages (n = 3) and error bars represent standard deviations. Strains include ΔSterne (open squares) and its lysogens obtained with either Wip1 (open diamonds in A, D, G, and J), Wip2 (open triangles in B, E, H, and K), Frp2 (open circles in C, F, I, and L) or Wip4 (cross-hatches in L). A Wip2-cured lysogen (star symbol in K) and a Wip2-cured lysogen re-infected with Wip2 (open circles in K) are included as well. Sporulating cultures were analyzed under the following conditions: (A–C) LD medium incubated at 30°C with agitation at 75 rpm; (D–F) LD medium incubated at 24°C with agitation at 75 rpm; (G–I) LD medium incubated at 24°C with no agitation; and (J–L) soil medium incubated at 24°C with agitation at 75 rpm.

A second distinct sporulation phenotype was also identified in this study. The entire group of Spo^+^ lysogens (Wip1, Wip2, Frp2, Slp1 and Wβ isolates) sporulated in LD cultures grown at 24°C with poor aeration, whereas the parental ΔSterne non-lysogen alone did not (Table 2). Furthermore, the parental phenotype (no sporulation at 24°C) was restored by phage curing. To investigate this phenotype, we looked at the sporulation kinetics of Wip1, Wip2 and Frp2 lysogens in conditions of varying temperature and aeration. In LD broth at 30°C with aeration at 75 rpm there was no real difference in the timing of sporulation for the lysogens and ΔSterne (Figure 3A–C). When, however, the growth temperature was 24°C, the Spo^+^ lysogens triggered sporulation at least four days earlier than ΔSterne and ultimately yielded much higher spore titers (Figure 3D–F). Without aeration this distinction was more profound and lysogens sporulated up to ten days earlier (Figure 3G–I) and by one year retained viabilities of >10^6^ spores and/or vegetative cells ml^−1^ while ΔSterne cultures were barely viable (<1×10^3^ cells ml^−1^). This enhanced sporulation phenotype was also observed in soil-extract medium (Figure 3J–L), but not in rich media like BHI or LB (data not shown).

Our findings show that lysogenic phages can manipulate the capacity of *B. anthracis* ΔSterne to sporulate. For several phages, lysogeny yields higher density and longer lived populations. For still other phages, the effect is to block sporulation; this inhibition is perhaps akin to capacity of the *B. anthracis* virulence plasmids to repress sporulation under certain conditions [35]. Interestingly, the Spo^−^ lysogens cannot be considered poor survivors compared to Spo^+^ forms, considering their long-term survival (>4 months) in soil-extract (Figure 3L).

### Lysogeny favors exopolysaccharide synthesis and multicellular behavior

**Figure 4:**
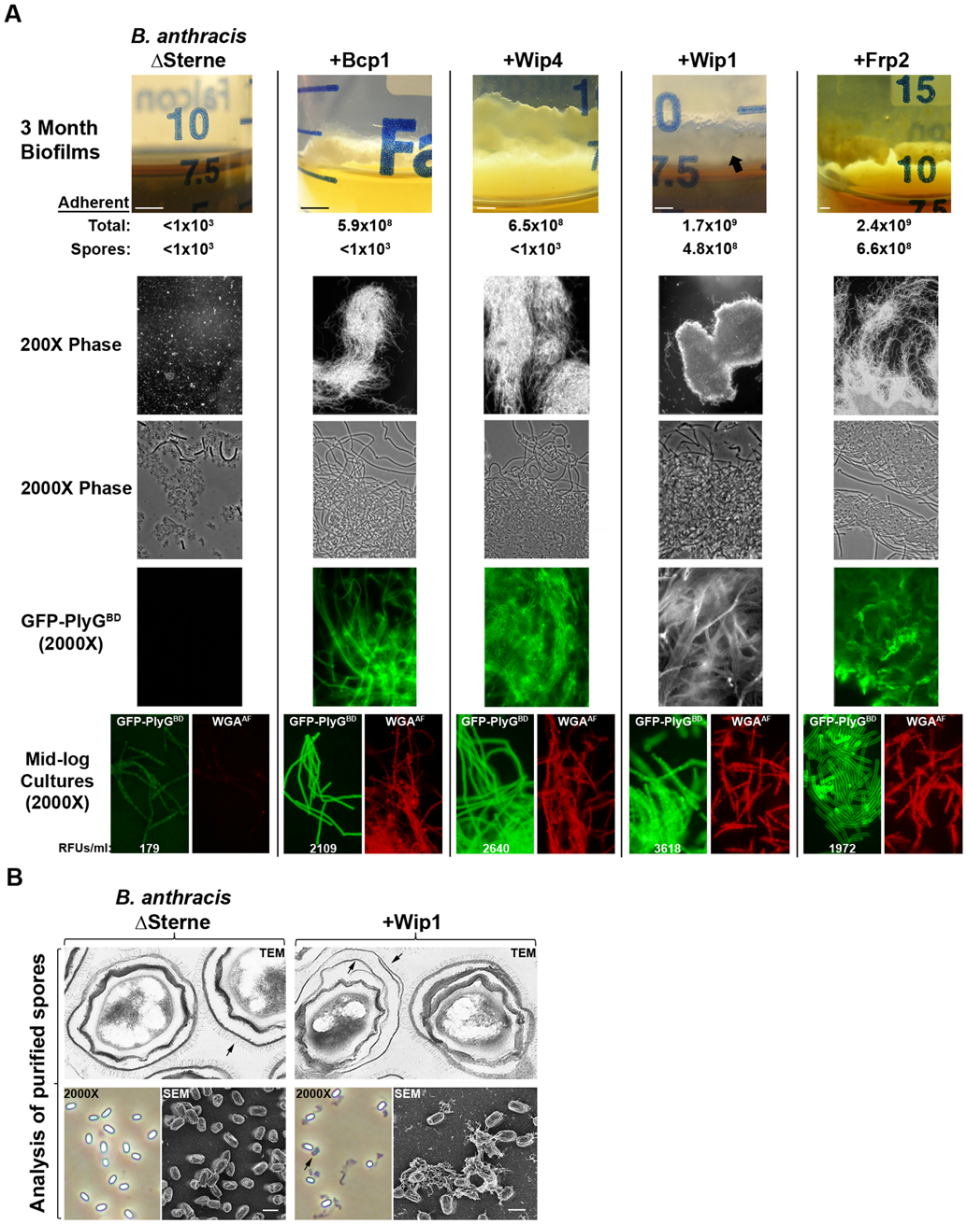
Phenotypic analysis of *B. anthracis* and its lysogens. (A) Biofilms formed at the liquid-air interface of 3 month BHI cultures grown without aeration at 24°C (scale bars are 0.5 cm). The total number of bacteria (vegetative cells and spores) and spores alone in each adherent biofilm were enumerated and visualized at 200X and 2000X magnification. GFP-PlyG^BD^-labeled cells are shown in 2000X fluorescence images taken with 0.3 second exposures. A non-colorized image is shown for the GFP-PlyG^BD^-labeled Wip1 lysogen. 2000X fluorescence images (0.3 second exposures) of mid-log phase BHI cultures labeled with GFP-PlyG^BD^ or WGA^AF^ are also shown. Relative fluorescence units (RFUs) corresponding to ∼1×10^8^ mid-log phase cells suspended in buffer are shown as averages of three experiments. (B) Lysogeny alters *B. anthracis* spore architecture. Transmission electron micrographs (TEM) show that the hallmark single-layer exosporium of ΔSterne actually consists of two distinct layers in >30% of Wip1 lysogen spores. Arrows show the surface nape structure for each exosporium. 45,000X magnifications are shown. Phase contrast images (2000X) show the Wip1 lysogen with an unusual surface structure indicated by an arrow. Closer inspection by scanning electron microscopy (SEM) reveals the surface structure to be an extended, grainy matrix (indicated by an arrow). Scale bars represent 1 µm.

The observation that Bcp1 lysogens form biofilm-like structures led to a more detailed analysis of multicellular behavior. Biofilms are the preferred environmental state for many organisms, consisting of complex and adherent multicellular assemblages maintained within exopolysaccharide-rich matrices [57]. Previously, *B. anthracis* strain Sterne (itself a φ20 lysogen [48]) was shown to form biofilms using an *in vitro* flow cell method [25]. Here, we used a modified version of a published protocol for biofilm formation [58], whereby BHI cultures were incubated for 3 months without aeration. Adherent biofilms at the liquid-air interface were recovered, plated for viability, and visualized by microscopy (Figure 4A). Here, ΔSterne yielded only poorly viable debris at the bottom of each culture tube, while the lysogens formed robust biofilms comprised of viable vegetative cells (for Spo^−^ lysogens Bcp1 and Wip4) or vegetative cell/spore mixtures (for Spo^+^ lysogens Wip1 and Frp2). By microscopy, the lysogen biofilms were dramatic bundles of parallel or convoluted vegetative filaments (of indeterminate length) in matrices bound by GFP-PlyG^BD^. GFP-PlyG^BD^ is a fluorescent *B. anthracis* exopolysaccharide-specific binding agent [46]. While the structure and biosynthetic pathway of this exopolysaccharide are known [59], [60], a role for this molecule in biofilm formation has not been previously described. Here, we have found a pronounced increase in the binding of GFP-PlyG^BD^ to the exopolysaccharide of all lysogens, based on microscopic and quantitative binding studies of mid-log phase bacteria labeled with GFP-PlyG^BD^ (Figure 4A). We also show this in parallel labeling studies with a second exopolysaccharide binding protein [61], Alexa Fluor-tagged wheat germ agglutinin. In conditions that support little surface-labeling of ΔSterne, there is a strong fluorescent signal for each lysogen (Figure 4A). The *B. anthracis* vegetative exopolysaccharide may thus promote the formation of biofilms in a manner akin to that previously described for the Eps exopolysaccharide of *B. subtilis* [62].

The Spo^+^ lysogens formed particularly complex biofilms. With ΔSterne/Wip1 as an example, the biofilms were heterogeneous with respect to observed cell-types. Extended bundles of vegetative cells were observed adjacent to regions of highly clumped spores and short vegetative rods, all contained in an exopolysaccharide-rich matrix (Figure 4A, Figure S3A). Interestingly, this biofilm was identical to that formed by either of two environmental *B. cereus* lysogens, RS423 and RS421, identified for this study (Figure S3C and S3D, Table S1). Both RS421 and RS423 formed adherent biofilms at 3 months, comprised of vegetative bundles, spores, and short rods in a matrix bound by GFP-PlyG^BD^.

The static, nutrient-poor, and long-term incubation conditions required to analyze biofilm formation here, could be favoring the accumulation of adaptive mutations in the host chromosome as previously described [63]. Thus, the long-term survival phenotypes observed here could reflect the cumulative effects of lysogeny and adaptive mutation. To examine this, we recovered ΔSterne::Wip2 lysogens from biofilms at 120 days and subjected them to heat-treatments that induced prophage loss. The resulting phage-cured derivative was then re-lysogenized with Wip2; both the cured and re-lysogenized forms were then examined in long-term survival assays. For the cured derivative, we observed complete reversion to the poor survival phenotype observed with ΔSterne (Figure 3K); thus, this strain did not encode mutations that alone promoted survival. For the re-lysogenized derivative, we observed survival kinetics identical to that of the original Wip2 lysogen (Figure 3K); thus, there was no adaptive mutation that enabled lysogen survival. The prophage alone is responsible for the long-term survival phenotype.

### Lysogeny favors changes in spore structure

The changes in vegetative cell structure observed here (i.e., filamentous bundles and increased exopolysaccharide) also led us to also investigate changes in spore structure. Spores were prepared from ΔSterne/Wip1 and subjected to an ultrastructural analysis that, indeed, identified two major changes (Figure 4B). First, thin-section TEMs revealed that over 31% of 500 ΔSterne/Wip1 spores had two exosporial layers; normally this is a single layer, with two layers appearing in <1% of ΔSterne spores. An unexplained double exosporium layer has previously been reported for *B. megaterium* [64] and *B. anthracis* (M. Fazzini, unpublished observations). The second major change concerned a novel external filamentous structure attached to each spore (Figure 4B). In SEMs, these structures are grainy matrices that appear to mediate adherence to other spores and the grid surface. These matrices did not bind GFP-PlyG^BD^ and were DNase-insensitive, thus they did not consist of the *B. anthracis* exopolysaccharide or DNA (data not shown).

### Phage-encoded RNA polymerase sigma factors drive phenotypic alterations

**Figure 5:**
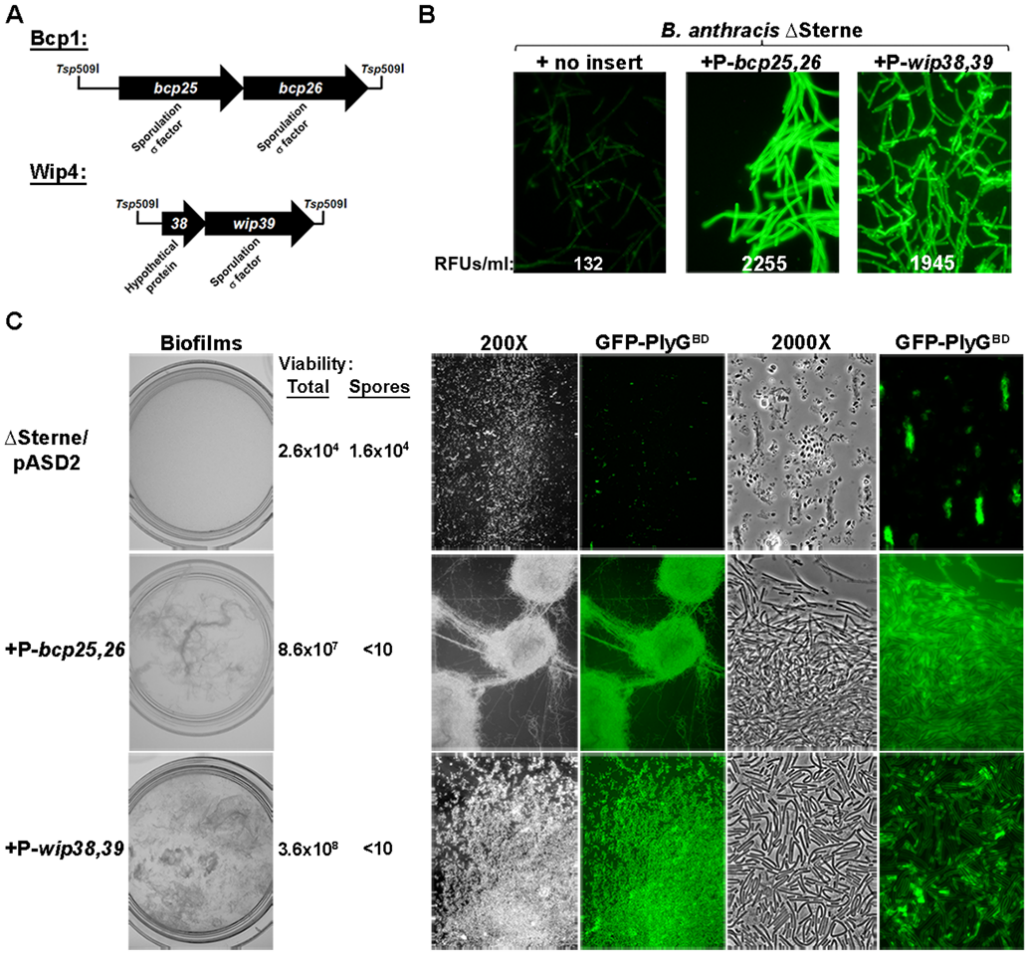
Phage-encoded effectors of phenotypic change in *B. anthracis*. (A) *Tsp*509I genomic fragments of Bcp1 (1,948 bp) and Wip4 (1,287 bp) identified in an expression library screen of phage-encoded loci that disrupt the Spo^+^ phenotype of ΔSterne. (B) GFP-PlyG^BD^-labeled mid-log-phages cells are shown in 2000X fluorescence images with 0.3 second exposures. ΔSterne/pASD2 derivatives are used with and without the indicated promoter-bearing Bcp1 and Wip4 loci. RFUs corresponding to ∼1×10^8^ mid-log phase cells suspended in buffer are displayed as averages of three experiments. (C) Biofilms formed at liquid-air interfaces of 3 ml LD cultures grown without aeration in 35 mm dishes for 1 week. Average numbers of organisms per ml are shown from three experiments for each condition, indicating total viability (vegetative cells and spores) and spore counts. For each strain, 200X and 2000X images of GFP-PlyG^BD^-labeled cells are shown with and without fluorescence emission.

We investigated the mechanism by which *B. anthracis* phages mediate phenotypic changes. Assuming that phage-encoded factor/s are responsible, we constructed and screened both Bcp1 and Wip4 genomic expression libraries for clones disrupting the Spo^+^ phenotype of ΔSterne on LD sporulation agar. Phage genomic fragments were generated by *Tsp*509I digestion and cloned into a self-replicating *B. anthracis* vector; after transformation into ΔSterne, the expression of library-encoded phage genes was driven only by their native promoters (i.e., no plasmid-encoded promoter were used). For each library, 0.5% of the resulting clones were Spo^−^ on LD plates, while <0.01% were Spo^−^ in vector-only controls. Sequence analysis revealed that each Bcp1 clone encoded the *bcp25*,*26* locus, while each Wip4 clone encoded *wip38*,*39* (Figure 5A). The deduced amino acid sequences of Bcp25, Bcp26, and Wip39 were very similar to that of several families of bacterial sigma factors, which are RNA polymerase subunits that confer promoter specificity to the transcription complex [65]. The strongest similarity was to σ^F^ and σ^G^ of *B. cereus s.l.* organisms, which are factors that direct sporulation gene expression [66]. Similarity to σ^B^, a sigma factor not involved in sporulation but nonetheless similar to σ^F^, was not detected. σ^F^ of *B. anthracis* Ames was 34%, 24%, and 33% identical to Bcp25, Bcp26, and Wip39, respectively (Figure S4). Wip38 was similar only to a hypothetical *B. anthracis* phage protein.

In the next series of experiments, we wanted to confirm that expression of *bcp25,26* and *wip38,39* was specifically driving the Spo^−^ phenotype. First, we used RT-PCR to show that these loci were indeed expressed from the Bcp1 and Wip4 prophages in ΔSterne during sporulation (Figure S5A and S5B). Next, the *bcp25,26* and *wip48,49* loci were taken out of the prophage context and cloned into plasmids as fragments that either included or excluded their respective 353-bp and 258-bp upstream promoters. After transformation of these vectors into ΔSterne, RT-PCR analysis confirmed that expression of *bcp25,26* and *wip38,39* in these backgrounds during sporulation still specifically required their respective upstream promoter sequences (Figure S5C and S5D). While the promoter-less *bcp25,26* or *wip48,49* clones sporulated at wild-type-like levels, the promoter-bearing clones (i.e., those expressing *bcp25,26* or *wip48,49*) (in the absence of any other phage components) were completely unable to sporulate (Table 2). These findings thus support a mechanism by which Bcp1- and Wip4-encoded sigma factors act alone (in the absence of other phage-encoded elements) to block sporulation. In agreement with this, we also found that insertional inactivation of either *bcp25* or *wip39* in the Bcp1 or Wip4 lysogen backgrounds, respectively, restores the Spo^+^ phenotype (Table 2) of these formerly Spo^−^ lysogens.

It was very interesting to find that *bcp25,26* and *wip38,39* were expressed during vegetative growth as well as sporulation (Figure S5E). For this reason, we investigated a role for these loci in changing vegetative cell phenotypes including exopolysaccharide and biofilm production. Ultimately, we found that expression of *bcp25,26* and *wip38,39* from their native promoters in pASD2 did specifically induce high-level, GFP-PlyG^BD^-mediated fluorescence in vegetative ΔSterne (Figure 5B). This exopolysaccharide expression was also accompanied by the formation of biofilms at the liquid-air interface of 1 week-old cultures (Figure 5C). Here, convoluted ropy masses and flaky, sheets of cells were observed, consisting of viable vegetative cells (and no spores) in a matrix bound by GFP-PlyG^BD^. The parental ΔSterne strain produced no biofilm whatsoever. Expression of *bcp25,26* and *wip38,39* therefore drives the vegetative phenotypes associated with Bcp1 and Wip4 infection.

We infer from these findings that Bcp1 and Wip4 prophages mediate host phenotypic modifications via sigma factors encoded by *bcp25,26* and *wip48,49*. While it is interesting that *B. anthracis* phages are using transcriptional regulatory proteins to effect host cell changes, it not surprising considering the pleiotropic effects such regulators should enable. Nonetheless, such a mechanism has been seen only for proteins like NucC and RecC of *Serratia marcescens* [67], [68], which are cryptic prophage-encoded transcriptional activators of extracellular nuclease and bacteriocin production. NucC and RecC are, however, not sigma factors, and roles for phage-encoded sigma factors have only been described in phage gene transcription [69], [70].

### Analysis of environmental *B. cereus s.l.* lysogens

The phenotypic changes associated with *B. anthracis* have to this point been based on analysis of lysogens generated in laboratory infections. In order to determine whether these changes also occur with naturally occurring lysogens, we examined environmental *B. cereus s.l.*-like strains bearing inducible prophages that are at least infective toward *B. anthracis*. We used the *B. cereus* strain ATCC 25621 (a cow feces isolate), and a group of three strains that were identified for this study including RS1045 (a worm gut strain), RS1255 (a human tonsil strain), and RS1557 (a fern rhizosphere strain). Each of these strains exhibited various *B. anthracis*-like genotypes and/or phenotypes (Table S1), and, in particular, was lysogenic for *B. anthracis* phages. These isolates most likely represent *B. cereus s.l.* members that are particularly closely related, genotypically, to *B. anthracis* [45], [71], [72], [73].

**Figure 6:**
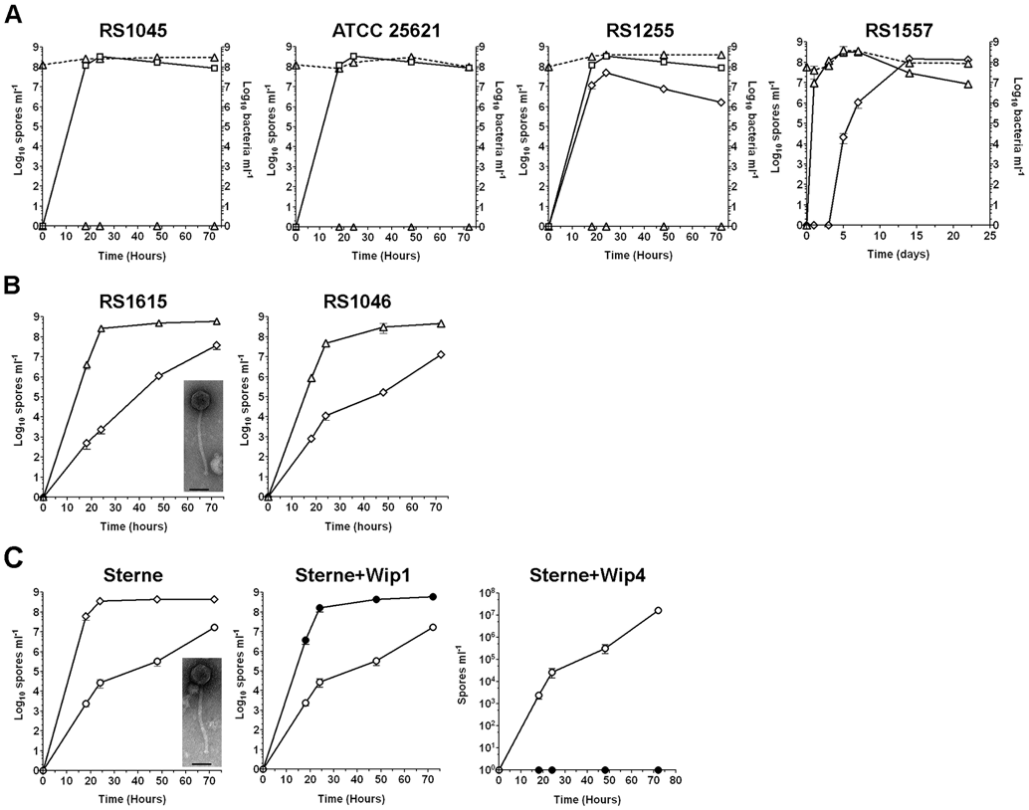
Sporulation phenotypes of natural *B. cereus s.l.* lysogens. Kinetic analyses of sporulation were performed in LD medium. All values are mean averages (n = 3) and error bars are standard deviations. (A) Sporulation of environmental *B. cereus*. Total viability (dashed lines) and spores alone (solid lines) are shown for growth conditions that include 37°C at 180 rpm (RS1045 and ATCC 25621), 30°C at 180 rpm (RS1255) or 24°C at 75 rpm (RS1557). *B. anthracis* ΔSterne (squares), the indicated environmental isolates (triangles), and phage-cured variants RS1255^CURED^ and RS1557^CURED^ (diamonds) are shown. (B) Sporulation for environmental *B. anthracis* strains. Cultures were grown at 37°C and aerated at 180 rpm. Strains include RS1615 or RS1046 (triangles) and RS1615^CURED^ or RS1046^CURED^ (diamonds). An electron micrograph of the inducible phage from RS1615 is shown (scale bar in 50 nm). (C) Sporulation for *B. anthracis* Sterne. Cultures were grown at 37°C and aerated at 180 rpm. Included are Sterne (circles), Sterne^CURED^ (diamonds), and the Wip1 or Wip4 lysogens of Sterne (closed circles). An electron micrograph shows the inducible Sterne phage (scale bar is 50 nm).

**Figure 7:**
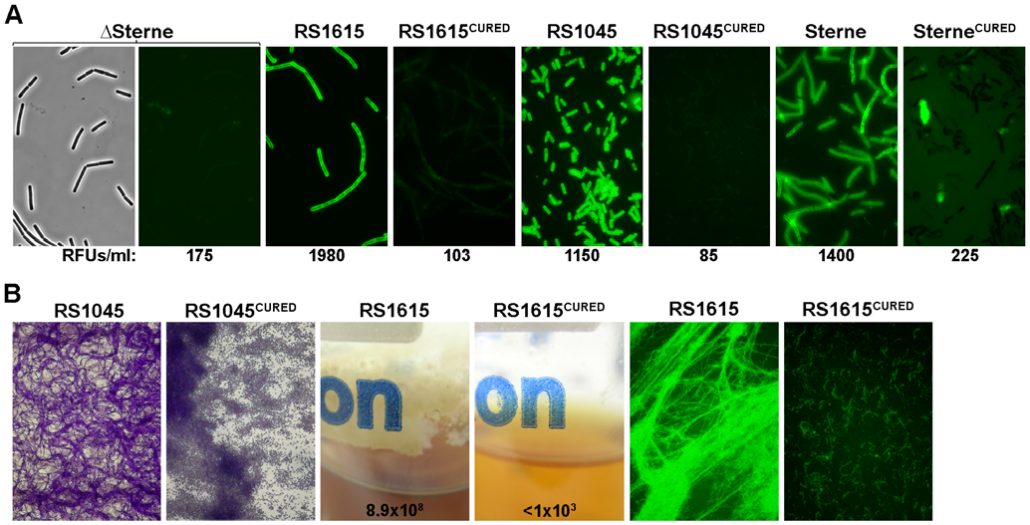
Exopolysaccharide and biofilm phenotypes of natural *B. cereus s.l.* lysogens. (A) Mid-log phase cells from BHI cultures labeled with GFP-PlyG^BD^. 2000X fluorescence images are 0.2 second exposures. For ΔSterne, a corresponding phase image is shown. RFUs for ∼1×10^8^ mid-log phase cells suspended in buffer are shown. (B) Biofilms formed by environmental *B. cereus* (RS1045) and *B. anthracis* (RS1615) backgrounds. For RS1045 strains, sections at the liquid-air interface of crystal violet-stained biofilms are shown. The RS1045 field is dominated by filamentous forms, while that of the phage-cured for is dominated by spores. Liquid-air interfaces are shown (along with total bacteria ml^−1^ for each culture) for the RS1615 strains. Additionally, 200X images of GFP-PlyG^BD^-stained biofilm (RS1615) or bottom-settled material (RS1615^CURED^) are shown.

Phenotypic analysis of ATCC 25621, RS1045, and RS1255 revealed that each strain was asporogenous in conditions that support sporulation of ΔSterne (Figure 6A). Focusing on RS1045, we also observed both the induction of high-level exopolysaccharide surface-expression and the formation of biofilms that were highly enriched for extended vegetative filaments (Figure 7A and 7B). For both the Spo^−^ defect of RS1255 and the exopolysaccharide and biofilm formation of RS1045, phage-curing was found to completely reverse the observed effect. Hence, RS1255^CURED^ could sporulate (Figure 6A) and RS1045^CURED^ produced a ΔSterne-like level of exopolysaccharide (Figure 7A) and yielded only adherent spores at the liquid-air interface of glass cover-slips (Figure 7B). Here, RS1045 and RS1255 are particularly interesting, since they are the strains from which Wip4 and Htp1, respectively, were originally isolated. The phenotypes associated with both Wip4 and Htp1 infection of their native *B. cereus* parental strains, therefore match that observed in experimental infections of *B. anthracis* ΔSterne seen in Table 2.

The Spo^+^ environmental strain RS1557 was also distinctive in that it displayed the “rapid” sporulation phenotype and sporulated well in conditions that caused RS1557^CURED^ to lag by several days (Figure 6A). The prophage of RS1557 is Frp2 (Table S1), which also induced the rapid sporulation of ΔSterne in Figure 3F. This finding, taken with that of the Spo^−^ environmental lysogens above, confirms that all the lysogeny-mediated phenotypes in ΔSterne can also be observed in environmental *B. cereus s.l.* lysogens, often with the same phages.

### Analysis of “natural” *B. anthracis* lysogens

To complete this phenotypic study, we next analyzed a group of naturally occurring *B. anthracis* lysogens. Included is the well-studied lab isolate Sterne, which harbored an inducible prophage (seen in Figure 6C) similar to that previously described [48]. The RS1615 and RS1046 environmental strains were also used, and were identified here as *bona fide B. anthracis* isolates based on the detection of chromosomal and/or virulence plasmid markers (Table S1). RS1615 is a soil isolate that encodes the *B. anthracis*-specific phage φ1615 (seen in Figure 6B), while RS1046 is a worm isolate that encodes Wip5 (seen in Figure 2E).

In a comparison of the sporulation kinetics of Sterne, RS1615, or RS1046 to that of their respective phage-cured derivatives (Figure 6B and 6C), the lysogens consistently sporulated faster and more efficiently (i.e., they yielded higher spore titers). These findings are particularly noteworthy, considering that well aerated cultures were used here (37°C and 180 rpm) which normally yielded no differences between ΔSterne and its Spo^+^ lysogens. Thus, for these *B. anthracis* lysogens, the prophage state induced a rapid sporulation phenotype. This phenotype, unlike that seen with the Spo^+^ ΔSterne lysogens, was observed in all conditions and was not temperature- and aeration-dependent. Further analyses focused on RS1615 and showed that both high-level exopolysaccharide expression (Figure 7A) and the formation of adherent biofilms enriched with filamentous bacteria (Figure 7B) was absolutely dependant on lysogeny.

The effect of poly-lysogeny was investigated using the Sterne strain. Sterne (a φ20 lysogen) was stably infected with either Wip1 or Wip4. The dominant phenotype of each poly-lysogen was found to be dictated by the new phage (Figure 6C). Thus, the φ20/Wip1 poly-lysogen had a rapid sporulation phenotype like that of ΔSterne/Wip1 in Figure 3D, and the φ20/Wip4 poly-lysogen was asporogenous like ΔSterne/Wip4 in Table 2. In all, these findings confirm that phenotypes observed in ΔSterne, are applicable to other *B. anthracis* backgrounds.

### *B. anthracis* lysogens colonize soil microcosms

Lysogeny of *B. anthracis* induces several phenotypes that should impact survival in the environment. Considering that *B. anthracis* is a member of a lineage of soil organisms, we proceeded to assess the impact of lysogeny on the ability of ΔSterne and environmental *B. anthracis* and *B. cereus* strains to survive long-term in conditions that mimic the soil.

**Figure 8:**
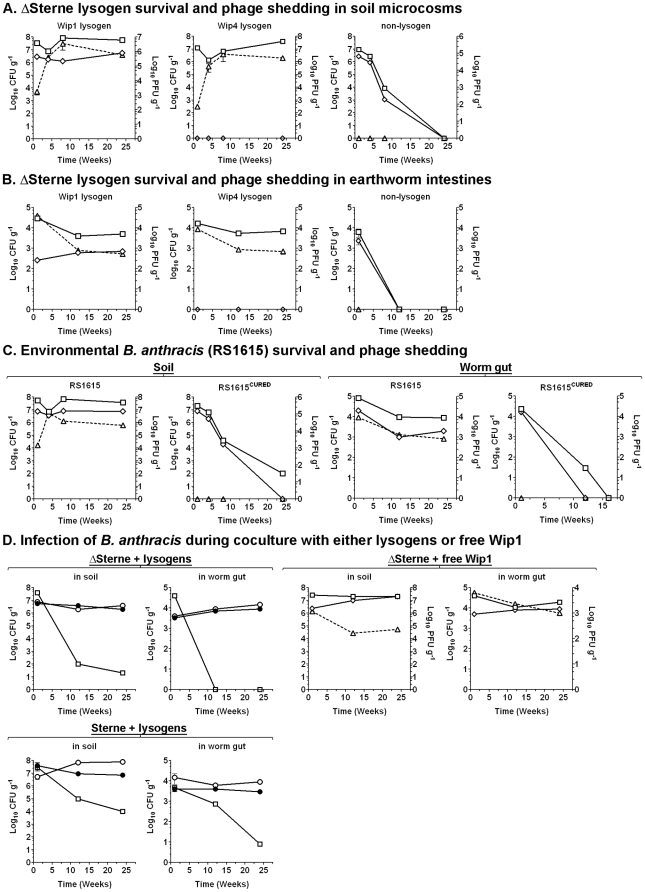
Impact of lysogeny on survival in soil and earthworms. Survival is shown as colony forming units (CFUs) per gram of recovered soil or worm guts (solid lines). Shed phages (dashed lines) are shown as plaque-forming units (PFUs) extracted per gram of soil or worm guts. Total *B. anthracis* viability (vegetative cells and spores; squares), spore counts (diamonds), and phages (triangles) are shown. Values are mean averages (n = 5) and error bars are standard deviations. (A) ΔSterne/pASD2 (non-lysogen) and its Wip1 and Wip4 lysogens in soil. (B) ΔSterne/pASD2 (non-lysogen) and its Wip1 and Wip4 lysogens in earthworm intestines. (C) Survival of environmental *B. anthracis* strain RS1615 and its phage-cured derivative (RS1615^CURED^) in soil and earthworm intestines. (D) Infection of *B. anthracis* during co-culture with lysogens or free Wip1 particles in soil or earthworm intestines. Strains ΔSterne/pASD2 and Sterne/pASD2 were either inoculated alone or with strains ΔSterne/Wip1 or RS1615 into each microcosm. At indicated times, ΔSterne/pASD2 and Sterne/pASD2 were recovered and scored by PCR for infection with Wip1 or φ1615. Survival of ΔSterne/pASD2 and Sterne/pASD2 inoculated alone (squares) and derivatives infected with Wip1 (closed circles) or φ1615 (open circles) are shown. For the spiking with Wip1, strain ΔSterne/pASD2 was inoculated alone or with 1×10^8^ Wip1 phages. Free Wip1 (triangles) is shown with total viability (squares) and spore counts (diamonds) for ΔSterne/pASD2 lysogenized with Wip1.

Microcosms were developed whereby ∼1×10^9^ vegetative bacteria (with <1×10^1^ spores) were inoculated into sterile potting soil, and at time points over 24 weeks were recovered and enumerated. For ΔSterne, viability of both Spo^+^ (Wip1, Slp1, and Wip2) and Spo^−^ (Wip4, Bcp1, and Frp1) lysogens remained around 10^7^ CFU g^−1^ of soil for 6 months (Figure 8A and Figure S6A). Environmental *B. anthracis* strains RS1615 and RS1046 were similarly durable (Figure 8C and Figure S6D), as was the environmental *B. cereus* strain RS1045 (Figure S6C). So the lysogens did exhibit long-term survival in artificial soil conditions. Indeed, the only requirement for survival was lysogeny, as ΔSterne, RS1615^CURED^, RS1046^CURED^, and RS1045^CURED^ yielded few or no viable organisms by 6 months (Figure 8A and 8C, Figure S6C and S6D). Microscopic analyses at 3 months showed the lysogens forming large exopolysaccharide-rich biofilms (Figure S7) akin to seen here in laboratory media. Thus, lysogeny-driven biofilm formation is closely associated with, and perhaps required for, long-term colonization of a soil habitat.

### *B. anthracis* lysogens colonize the gut of *Eisenia fetida*, the invertebrate redworm

As part of his seminal studies into the cause of infectious disease in animals, Louis Pasteur noted an abundance of earthworms at buried anthrax carcasses and proceeded to identify *B. anthracis* spores in the guts of these worms [53]. While he postulated that earthworms were important in the *B. anthracis* lifecycle, there has been no further study of this interaction. Nevertheless, the worm gut is a complex microbial habitat [74] that includes *B. cereus* and *B. thuringiensis* [7], [75]. These are the reasons why we initially looked for *B. anthracis* phages in the worm gut and why we next proceeded to study colonization.

Earthworm microcosms were developed based on a combination of published protocols [75], [76] whereby non-sterile soil containing individual animals were inoculated with ∼5×10^9^ bacteria and incubated for 1 week, prior to 22 subsequent weeks marked by repeated passages through “clean” soils (i.e., no *B. anthracis*). At 1, 12, and 24 week time points after inoculation into clean soil, gut contents were plated on selective media to enumerate *B. anthracis* derivatives. For ΔSterne, the viability of both Spo^+^ (Wip1, Slp1, and Wip2) and Spo^−^ (Wip4, Bcp1, and Frp1) lysogens remained steady around 10^4^ CFUs g^−1^ of worm gut over 24 weeks (Figure 8B and Figure S6B). The *B. anthracis* strains RS1615 and RS1046 and *B. cereus* strain RS1045 were stably recovered as well (Figure 8C and Figure S6D). For strains that lacked lyosgenic phages, including ΔSterne, RS1615^CURED^, RS1046^CURED^, and RS1045^CURED^, only transient colonization was noted with few recoverable bacilli at 24 weeks. *B. anthracis* therefore colonizes the intestinal tract of earthworms in a process that requires lysogeny. Presumably, latent endosymbiotic capabilities of *B. anthracis* are being activated in the worm gut.

### Phage shedding in environmental microcosms

Bacteriophage-host interactions are often viewed only in terms of lytic behavior and integrated prophages (i.e., lysogeny). Nonetheless, a complicated range of other conditions may also exist, including chronic states marked by phage budding or extrusion and persistent infections marked by episomal prophages, low-level bacterial lysis, phage shedding, and prophage curing [77]. Such behaviors are influenced by growth conditions and other factors, and are believed to be common for environmental lysogens.

Here, we investigated the viral-host state in *B. anthracis* and found evidence of pseudolysogeny. Pseudolysogeny occurs when phages multiply in a small fraction of a population, shedding infective particles, and maintaining environmental levels of virus in the presence of infected bacterial populations [77], [78], [79]. In studies of the 24 week soil and earthworm microcosms, we observed considerable viral shedding by otherwise stable *B. anthracis* and *B. cereus* lysogen populations (Figure 8A–C, Figure S6A–D). For each lysogen, we observed roughly the same number of free phages as viable bacteria in each microcosm throughout the experiment. No PFUs were observed with non-lysogenic strains. The shedding of phage here is consistent with pseudolysogeny. We similarly checked shedding in sheep blood, BHI, and LD media and observed free phages released from most lysogens, with the exception of Wip1, which only shed in blood.

### Lysogeny and the acquisition of novel traits in environmental microcosms

The shedding of phages by *B. anthracis* and *B. cereus* lysogens could reflect a mechanism for DNA transfer (and thus niche expansion) among these organisms. To investigate this, a series of co-cultures were established in soil microcosms and worms, in which ΔSterne or Sterne strains (transformed with pASD2 to provide antibiotic resistance and enable selective recovery) were co-inoculated with *B. anthracis* and *B. cereus* phage “donor” strains. Phages shed by the donors may infect the susceptible ΔSterne or Sterne “recipients”, which can then be selectively recovered and analyzed. Identical experiments were also established whereby free Wip1 phages were used in lieu of a donor lysogen.

For the soil microcosms, recipient ΔSterne/pASD2 or Sterne/pASD2 organisms were inoculated one day before the addition of “donor” lysogens or phages. The recipients were recovered at indicated time points, enumerated, and examined to confirm lysogeny. When ΔSterne/pASD2 was incubated without a phage source as a control, little or no survival was apparent at 24 weeks (Figure 8D, Figure S6E). This effect is not as severe with Sterne/pASD2 alone, possibly because it is a φ20 lysogen [48] which has been heavily passaged in the laboratory. Nonetheless, when ΔSterne/pASD2 or Sterne/pASD2 were co-cultured with any of the donor lysogens (or free phage), there was a pronounced improvement in survival, with 10^6^–10^8^ CFUs detected per gram of soil (Figure 8D, Figure S6E) over the experiment. In each case, the surviving recipient strains had all become lysogenized with Wip1, φ1615, φ1046, or Wip4. Thus, *B. anthracis* phages that are either shed or added as free particles can lysogenize susceptible strains and confer the long-term survival phenotype in the soil milieu.

For the worm experiments, “donor” lysogens (ΔSterne/Wip1, RS1615, RS1046, or RS1045) were used to colonize the worm gut for one week, before the animals were recovered, washed, and passaged through clean soil (i.e., no added bacteria). Again, ∼1×10^8^ free Wip1 particles were added in lieu of the donor lysogens in some experiments. After one week, the worms were moved to new microcosms and exposed to “recipient” bacilli before they were again recovered, washed, passaged for 24 weeks through clean soil, and plated for enumeration. For ΔSterne/pASD2 and Sterne/pASD2 incubated without a phage source as a control, few if any organisms were recovered from the worm gut at 24 weeks (Figure 8D). In the presence of either the lysogens or free phages, however, persistent survival of 10^3^–10^4^ CFUs per gram of worm gut was routinely observed, including both vegetative and spore forms. In each case, recipient strains had all become stable lysogens of Wip1, φ1615, φ1046, or Wip4. Phages shed by stable worm gut populations of *B. anthracis* can, therefore, infect incoming non-lysogens and confer the novel worm-survival phenotype.

Having observed phage shedding and concomitant lysogeny in soil and worm microcosm co-cultures, we proceeded to a similar analysis in other environments that support phage shedding. Sheep blood, BHI and LD cultures were all co-inoculated with donor lysogens and the ΔSterne/pASD2 recipient. Recipient strains were recovered after 2 days and examined to confirm lysogeny. In most cases where we previously observed viral shedding (Figure S8), infection of the recipient strain was detected (Table S5). The exception was Wβ, which is shed in all conditions, yet only lysogenizes ΔSterne/pASD2 in the sheep blood.

### Phage-induced expression of host loci in the soil and earthworm intestine

To understand how prophages activate a phenotype like worm gut colonization, we sought *B. anthracis* loci expressed in this milieu. Two methods were used to identify bacterial promoters that, when fused to a GFP gene, yield colonies that are: 1) fluorescent in a Wip4 lysogen on worm-extract agar; 2) non-fluorescent in ΔSterne (non-lysogen) on worm-extract agar; and 3) non-fluorescent in a Wip4 lysogen on BHI agar. This differential regulation should reflect a gene that is induced by virtue of lysogeny in the worm gut.

**Figure 9:**
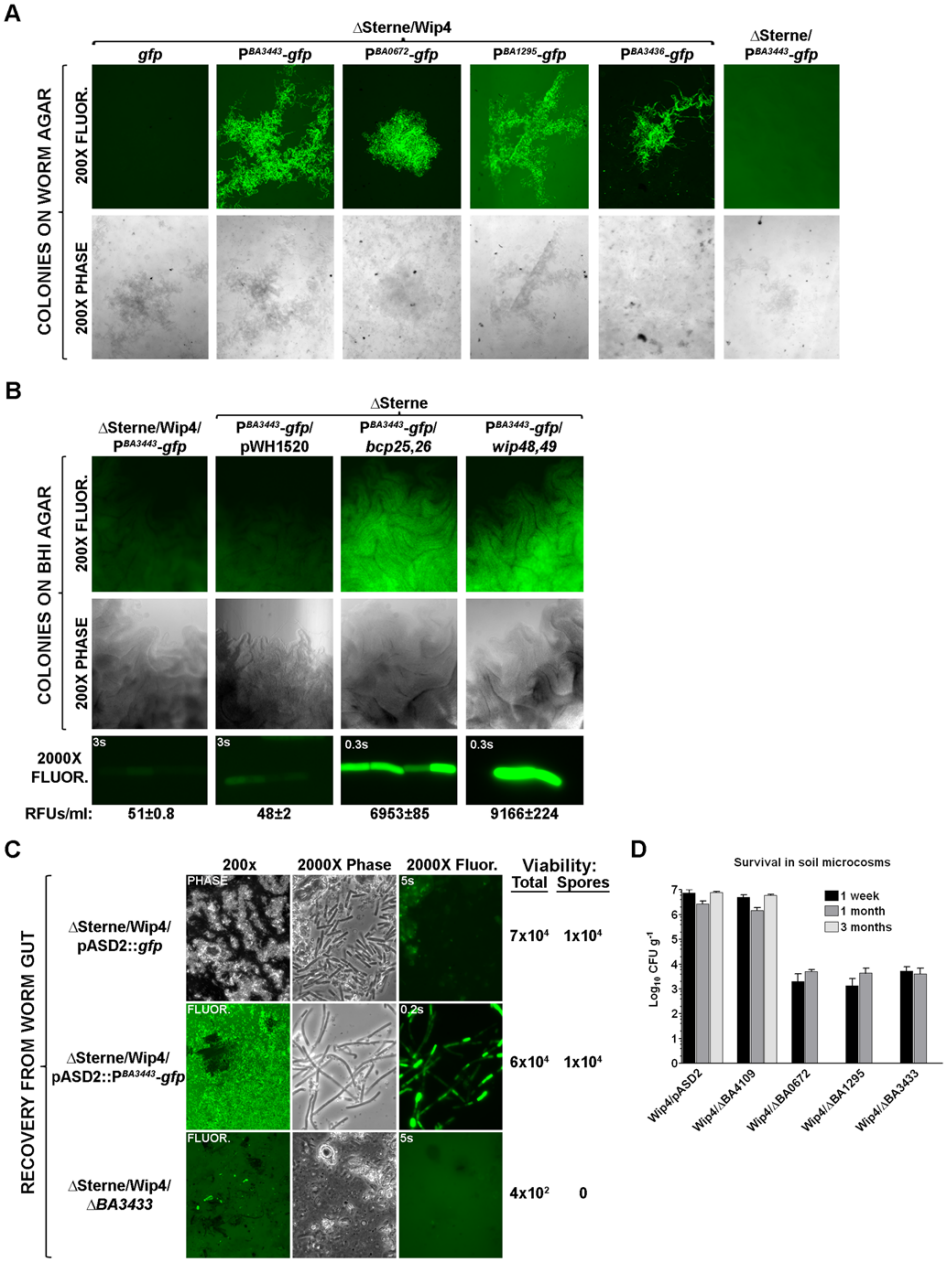
Phage-regulated loci in *B. anthracis*. (A) Promoter-*gfp* fusions expressed on worm agar. Bacteria were plated for 16 hours at 30°C and resulting colonies are shown in 200X phase-contrast and fluorescence images (0.5 second exposures). Strains are ΔSterne or its Wip4 lysogen (“/Wip4”) transformed with pASD2 encoding *gfp* alone or indicated promoter fusions (P). (B) Expression of P*^BA3443^*-*gfp* on BHI agar in either the ΔSterne/Wip4 background or ΔSterne co-transformed with the compatible vector pWH1520 (either without and insert or that bearing *bcp25,26* or *wip48,49*). Bacteria were plated for 16 hours at 30°C and colonies are shown in 200X phase-contrast or fluorescence images with 0.5 second exposures. 2000X fluorescence images of representative bacteria are shown with exposure times. RFUs are averages of three experiments for ∼1×10^8^ mid-log phase cells grown in BHI and suspended in buffer. (C) *BA3443* and earthworm colonization. One month after colonization, bacteria were recovered, visualized, and enumerated. Strains are ΔSterne/Wip4 transformed with indicated vectors or the ΔSterne/Wip4 mutant Δ*BA3443*. Hind-guts were examined by phase-contrast or fluorescence microscopy at 200X or 2000X magnification. Exposure times are indicated in 2000X images. Average CFUs per gram of gut is shown from three experiments, indicating total viability (vegetative cells and spores) and spores alone. (D) *B. anthracis*-encoded loci required in the soil. Bacteria were recovered and enumerated at the indicated times from inoculated soil microcosms. Strains include ΔSterne/Wip4 with pASD2 alone or the indicated mutations. Values are mean averages (n = 3) and error bars are standard deviations.

For one method we constructed a promoter-probe library by fusing ΔSterne genomic fragments to a promoterless GFP gene. Ultimately, only one fusion exhibited differential regulation – fluorescent in a Wip4 lysogen on worm agar (Figure 9A) but not BHI agar (data not shown) and non-fluorescent in a ΔSterne background in any condition (data not shown). Sequence analysis revealed the promoter of *BA3436*. BA3436 is a 317 residue protein that is >40% identical to luciferase proteins of Gram-positive and Gram-negative bacteria (Conserved Domain Database entry cd01096), and is expressed as the promoter-proximal gene of a likely tricistronic locus that includes *BA3435* and *BA3434*.

Three additional differentially regulated promoters were identified based only on the predicted functions of their ORFs. The presence of *BA3443*, *BA0672*, and *BA1295* in the *B. anthracis* genome has previously been described as a remnant of ancestral endosymbiotic or insect-pathogenic lifestyles [30]. BA3443 is a homolog of the enhancin metalloprotease family (Pfam entry 03272), that includes many *B. cereus s.l.* proteins with predicted roles in mucus degradation and gut wall interactions with invertebrates [80]. BA0672 and BA1295 are homologs of the immune inhibitor A metalloprotease family (Pfam entry 05547) that includes several *B. cereus s.l.* proteins with predicted roles in degrading antibacterial peptides and insect gut survival [81]. As these proteins could mediate worm-gut survival for *B. anthracis*, we created transcriptional fusions of *BA3443*, *BA0672*, and *BA1295* to *gfpmut2* and screened them for differential fluorescence in a ΔSterne/Wip4 background. Using *BA3443* as an example here, the promoters of all three loci directed GFP-mediated fluorescence in bacterial colonies on worm agar (Figure 9A), but not BHI agar (Figure 9B). In a ΔSterne background, no fluorescence was observed on worm agar (Figure 9A). Thus, *BA3443*, *BA0672*, and *BA1295* are specifically induced in the worm milieu in lysogen-dependent manner.

Remaining with *BA3443* as the model for phage-induced genes here, we sought to determine whether the *bcp25,26* and *wip48,49* products could drive this up-regulation. Promoterless versions of *bcp25,26* and *wip48,49* were cloned into pWH1520 and introduced into ΔSterne also harboring plasmid pASD2::P^BA3433^-*gfp*. Resulting strains were plated on BHI agar, which is a condition that does not normally support *BA3443*-directed GFP fluorescence in either the ΔSterne or ΔSterne/pWH150 backgrounds (Figure 9B). When *bcp25,26* or *wip48,49* was present and expressed from pWH1520, we saw brightly fluorescent colonies on BHI agar that also yielded over 100-fold higher RFU levels (compared to controls) in BHI liquid (Figure 9B). *BA3443* expression is therefore phage gene-dependant. This effect is likely to reflect a mechanism whereby phage-encoded sigma factors (including Bcp25, Bcp26, and Wip49) direct RNA polymerase to host promoters like that of *BA3443*.

Next, we confirmed that expression of *BA3443* does occur in the worm gut. Worms were colonized for one month with ΔSterne/Wip4 lysogens encoding *gfp* alone or that fused to the *BA3443* promoter. Hind guts were extracted and plated for viability to first confirm colonization with both strains (Figure 9C). Next, gut contents were examined microscopically and found to contain abundant masses of vegetative cells that were highly fluorescent only for the P^BA3443^-*gfp*-bearing strain. The *BA3443* locus is therefore expressed by *B. anthracis* lysogens colonizing the worm gut.

Finally, we evaluated the importance of these phage-induced genes in the colonization process. Toward this end, we insertionally inactivated *BA3443*, *BA0672*, and *BA1295* in a ΔSterne/Wip4 background and inoculated each mutant into the worm microcosm. After 1 month, only the *ΔBA3443* mutant exhibited a severe defect and was barely recoverable (Figure 9C and data not shown). Since *BA0672* and *BA1295* encode immune inhibitor A homologs that are 67% identical to each other, they may be redundant activities in the worm gut. To see if these findings extend to soil-survival conditions, we similarly examined each mutant in 3 month soil microcosms. While the control strains (with either pASD2 or a *ΔBA4109* mutation) survived to 3 months, the *BA3443*, *BA0672*, and *BA1295* mutants were only transiently detected and therefore unable to survive (Figure 9D). These findings confirm that phage-regulated *B. anthracis* loci are required for long-term colonization of the soil and earthworm gut.

## Discussion

Anthrax is a disease of antiquity that continues to pose a threat as a biological weapon. While the pathogenesis of anthrax is well understood, surprisingly little is known regarding how the causative agent, *B. anthracis*, completes its lifecycle in the environment between outbreaks. Thus, an important biological question remains.

The lifecycle of *B. anthracis* is often described by short vegetative bursts in infected hosts alternating with long periods of dormancy as an environmental spore until disease is re-established. Environmental surveys show that *B. anthracis* can sporulate at anthrax carcasses, yielding an infectious cell type that is resistant to adverse conditions and is recoverable from the soil for long periods. Nonetheless, these surveys also present conflicting results that hint of alternative lifestyles for *B. anthracis* based on its vegetative form. First, spore contaminations may not diminish in spite of environments that should disperse spore populations (i.e., sporulating vegetative cells replenish the reservoir). Second, sporulation rates at anthrax carcasses can be low, and spore counts at such sites and throughout enzootic areas insufficient to explain outbreak cycles (i.e., there is a vegetative reservoir). These findings are in line with both Van Ness' hypothesis that vegetative incubator areas exist in the environment [24] and the finding of Saile and Koehler that vegetative growth may occur in the rhizosphere [27].

To investigate alternate environmental behaviors for *B. anthracis*, we considered a role for bacteriophages based on their well described contributions to bacterial adaptive behavior and niche expansion. During anthrax infections, rapidly growing vegetative cells likely represent a single clone, thus the population should not be exposed to exogenous phages. Rather, the staggering numbers of bacilli released after host death, ranging from 10^7^ to 10^9^ ml^−1^ of blood, should certainly be the targets for the variety of *B. anthracis*-active phages found throughout soil and water samples in enzootic locations. While environmental *B. anthracis* could be a source of such phages, *B. anthracis*-like *B. cereus* and *B. thuringiensis* strains (encoding *B. anthracis*-infective phages) are often ubiquitous at enzootic areas [82] and could be phage donors. The fact that so many environmental and laboratory *B. anthracis* isolates are lysogenized with a range of inducible phages suggests that environmental infection does occur. How this infection impacts *B. anthracis* is the subject of this study.

First we collected *B. anthracis*-specific bacteriophages from the soil (specifically from worm castings, potting material, and landfill samples), human tonsil, fern rhizosphere, and earthworm gut. These environments yielded free plaque-forming units and fosfomycin-induced phages from *B. cereus s.l*.-like organisms (including two *bona fide B. anthracis* strains). The earthworm gut was a particularly rich source, providing at least five phages from worms recovered at two distinct geographical locations.

The analysis of lysogeny was initiated in a ΔSterne background infected with phages from the soil, rhizosphere, tonsil and worm gut. Sporulation phenotypes divided resulting derivatives into two classes, including an asporogenous group (the Wip4, Wip5, Frp1, Htp1, and Bcp1 lysogens) and another with a rapid sporulation phenotype in low growth temperatures/aeration (the Wip1, Wip2, Frp2, and Slp1 lysogens). Both classes nonetheless survived long-term in culture (>3 months) by producing biofilms comprised of pronounced vegetative growths highly enriched with *B. anthracis* exopolysaccharide. High-level exopolysaccharide expression was a phenotype specific to the lysogens, even during exponential growth, and is possibly required for biofilm formation. Within each biofilm, vegetative cells exhibited a multicellular or rhizoidal phenotype, distinct from the shorter rod-shaped form seen during growth. A switch to multicellular behavior has also been reported for biofilms of other *Bacillus* species [62], as well as *B. cereus* adhering to the gut wall of insects [83], [84] and *B. anthracis* growing in a model rhizosphere system [27]. For the Spo^+^ lysogens, clumps of spores were also apparent throughout each biofilm. Ultrastructural analysis of Wip1 lysogen spores revealed two major changes including a double exosporium and the elaboration of an extracellular matrix which could favors interactions among spores in the biofilm.

The prophage-induced changes in sporulation, exopolysaccharide, and biofilm phenotypes suggest an impact on environmental survival. For this reason, we examined the ΔSterne lysogens in soil microcosms and *Eisenia fetida* intestines. Regardless of the environment, all of the lysogenic derivatives tested survived up to six months while the parental ΔSterne strain declined steadily from the outset. In the soil, survival involved both high-level exopolysaccharide expression and the formation of biofilms. In the worm gut, the numbers of recovered lysogens were likely an underestimation based on the appearance of vast numbers in the microscopic analysis of Figure 9C. Most bacterial organisms entering the worm intestine are either digested or passed out in castings [85]; colonizing organisms must adhere tightly to the gut wall and thus may be difficult to dissociate and enumerate. For *B. cereus*, a tight interaction is seen with long bacterial filaments that adhere to the invertebrate gut wall and shed spores and perhaps smaller rods into the intestinal lumen from their distal ends [84]. This may be the situation for *B. anthracis*. Our findings that some Spo^+^ *B. anthracis* lysogens can sporulate under conditions of poor aeration may mark an adaptation to the earthworm gut, an extremely anoxic environment [74].

The phenotypic changes observed in *B. anthracis* ΔSterne were confirmed to varying degrees in three additional background types, including, 1) the *B. anthracis*-like *B. cereus* strains RS1045, RS1255, RS421, RS423, and RS1557; 2) *B. anthracis* Sterne; and 3) environmental *B. anthracis* strains RS1615 and RS1046. Each of these strains is a naturally occurring lysogen, and most phenotypic comparisons were made to phage-cured derivatives of these strains. With respect to the *B. anthracis* virulence plasmids, we used variants that had no plasmids (ΔSterne and RS1046), one plasmid (Sterne), or was PCR-positive for virulence genes of two plasmids (RS1615). With this diverse set of *B. anthracis* and *B. cereus* strains a common set of phenotypes was seen that include sporulation inhibition, rapid sporulation, exopolysaccharide production, biofilm formation, long-term survival in soil microcosms, and earthworm gut colonization. Phages like Wip4, Frp2, Htp1, and Wip5 were notable in that they exerted the same phenotypes in ΔSterne as they did in the strains from which they were originally isolated (RS1045, RS1557, RS1255 and RS1046, respectively). These findings thus confirm that lysogeny of *B. anthracis* is associated with major phenotypic changes and the acquisition of at least two new niches in the laboratory.

The mechanism by which *B. anthracis* prophages specifically induce phenotypic changes likely requires both phage- and host-encoded loci. To find these factors and determine how lysogen conversion proceeds, we first screened the genomes of Wip4 and Bcp1 for loci that disrupt the sporulation phenotype of ΔSterne. The lysogen converting functions of *bcp25,26* and *wip38,39* were thus identified, revealing a process that requires phage-encoded RNA polymerase sigma factors. Not only was expression of these sigma factors required for the asporogenous phenotype, but for exopolysaccharide production and biofilm formation as well. Considering the range of phenotypic changes observed here, it should not be surprising that phage-encoded transcriptional regulatory proteins are the responsible effectors. Sigma factors may represent a very efficient means for “foreign” phage DNA to manipulate *B. anthracis* and induce otherwise latent phenotypes. The extent of these effects and whether they are induced by sigma factors of phages other than Wip4 and Bcp1 will be investigated. Bacterial sigma factors and other transcriptional regulatory molecules are encoded throughout the genomes of *B. anthracis* phages [46], [70]. Interestingly, there has been no previous description of sigma factors with lysogen converting functions.

We identified four host-encoded loci that were transcriptionally active in a ΔSterne/Wip4 background in the worm milieu. These loci encoded proteins involved in environmental signaling and social groupings (luciferase), the degradation of intestinal mucus and colonization of invertebrate guts (enhancin), and survival in the invertebrate gut (immune inhibitor A). The BA3443 enhancin was actually required for colonization of the worm gut (and soil) here, while the immune inhibitor A homologs BA0672 and BA1295 were individually required only for soil. Expression of *BA3443* was observed in the worm gut and was found to be dependent on either *bcp25,26* or *wip38,39*. These findings support the mechanism by which phage-encoded sigma factors, like those of *bcp25,26* and *wip38,39* drive the expression of host genes that encode the means to colonize the worm gut. Elucidation of such host genes in the manner described here, followed by mutational analyses, and microscopic studies of interactions in the worm gut will ultimately be needed to decipher this colonization process.

Whether or not there is environmental evidence of lysogen-mediated vegetative survival awaits surveys of enzootic areas, with particular attention to *B. anthracis* cell-types recovered from earthworm and rhizosphere samples, and the free phage and prophage content of such environments. Nonetheless, there is already some evidence that phages do impact *B. anthracis*. Despite the highly monomorphic nature of global *B. anthracis* populations, phenotypic diversity has been described in relation to strain ecology and distribution [86]. Genotypic groups, endemic to particular areas, can vary with respect to colony morphology and tenacity, chain length, phage susceptibility, and the kinetics of sporulation [1], [86]. While the genetic basis for these differences was not investigated, these are features that may vary with lysogeny. Several mechanisms exist for genetic variation in *B. anthracis*, including natural mutation and the mutagenic effects of nitration from the host inflammatory process [43]. Recently, another source of variation was also proposed whereby bacteriophages drive the emergence of *B. anthracis* derivatives with altered genotypes and phenotypes. Here, Kiel et al. [43] identified a *B. anthracis* Sterne strain, from a mixed population including several known *B. anthracis* and *B. cereus* strains, that was likely infected with a phage shed by *B. cereus* in that mixture. Using a molecular genotyping method to assess 15 variable-number-tandem-repeat markers, they found that the infected strain had not only become genotypically distinct from Sterne, but also from all known *B. anthracis* isolates. If bacteriophages can drive the appearance of *B. anthracis* strains that are not recognized as such, interesting environmental lysogens could be misidentified and underestimated.

**Figure 10:**
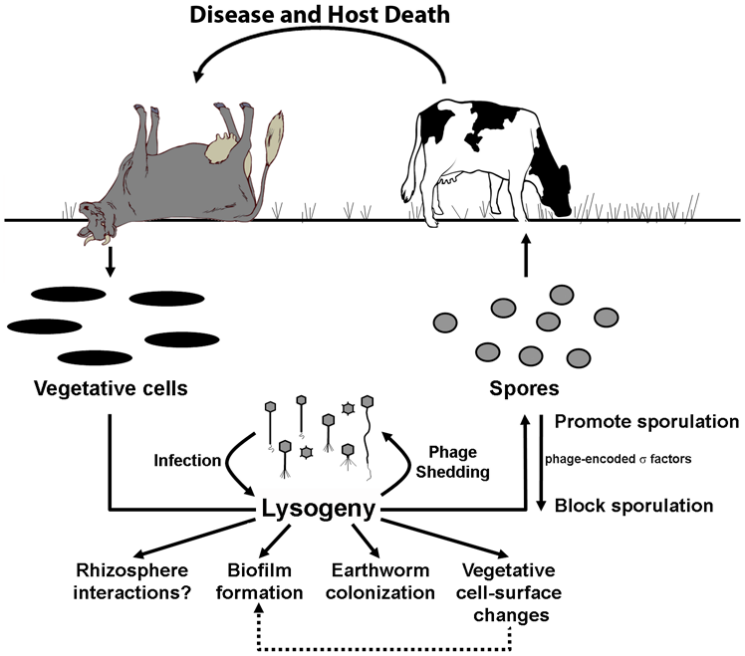
Alternate lifestyles for *B. anthracis* in the environment? Solid arrows trace a lifestyle in which there are alternatives to starvation and sporulation driven by lysogeny. By infecting an otherwise non-lysogenic *B. anthracis* strain with several distinct phages, we have observed changes in sporulation, exopolysaccharide and biofilm formation, soil survival, and earthworm colonization. Such phenotypic changes may favor saprophytic or endosymbiotic lifestyles in the soil.

Our results provide the first indication of a potential pivotal role for bacteriophages in the *B. anthracis* lifecycle. Infection drives a series of changes, with respect to morphology, sporulation, exopolysaccharide, and biofilm phenotypes, which could affect environmental functioning and reflect behaviors required for soil and/or invertebrate intestinal survival that we observed. Exactly how these phenotypes mediate survival remains to be determined. However, we can speculate that biofilms (mediated by exopolysaccharide production) could maintain vegetative reservoirs that can interact with and ultimately colonize grazing invertebrate worms. The filamentous growth may reflect that seen with bacilli attached to walls of insect guts [83], [84], where smaller rods and spores are shed into this anoxic environment and ultimately out of the worm. Inhibition of sporulation could enable faster growth in the competitive microbial environment in the worm gut. Regardless of how colonization proceeds, the shedding of bacteriophage from such populations is envisioned as seeding the environment with particles that, in turn, infect local susceptible bacilli and enable expansion into that niche. The fact that environmental *B. anthracis* isolates have such varied inducible prophages that enable the phenotypic changes described here, supports the relevance of our assertions. It seems, therefore, that rather than the bleak prospect of dormancy, *B. anthracis* may be capable of the dynamic, alternative lifestyle shown in Figure 10. Further studies will focus on areas ranging from phage-encoded factors that direct host-gene expression, to how those host genes elicit phenotypic changes, and ultimately to how lysogens interact with environments such as the earthworm gut. In this manner, we will continue study of the process by which *B. anthracis*, survives outside its animal hosts.

## Materials and Methods

### Bacterial strains and plasmid constructs

The *B. anthracis* strains used in this study are described in Table S6. Sources of environmental *B. anthracis* and *B. cereus* strains are listed in Table S1. Plasmid pWH1520 is an *E. coli*-*Bacillus* spp. shuttle vector. Plasmid pASD2 is an *E. coli*-*B. anthracis* shuttle vector with a temperature-sensitive replicon that cannot support plasmid replication at growth temperatures above 37°C [87]. For construction of the pASD2::*gfp* transcriptional fusion vector, *gfpmut2* [88] was amplified by PCR with indicated primers (Table S7) and the product was cloned into the *Kpn*I-*Sma*I sites of pASD2; this creates a unique primer-encoded *Eco*RI site upstream of *gfpmut2* for promoter-probe fusions. Promoter-bearing sequences were amplified by PCR with the indicated primers to generate a 543-bp fragment that encoded 444-bp of the *BA3443* promoter, an 881-bp fragment that encoded 740-bp of the *BA0672* promoter, a 992-bp fragment of that encoded 147-bp of the *BA1295* promoter, and a 444-bp fragment that encoded 356-bp of the *BA3436* promoter. For insertional mutagenesis, internal fragments of *BA3443*, *BA0672*, *BA1295* and *BA4109* were amplified by PCR with indicated primers and cloned into the *Kpn*I site of pASD2. For expression studies, the *bcp25,26* locus was PCR-amplified from a Bcp1 DNA template with indicated primers yielding either a 1948-bp fragment that includes 353-bp of promoter sequence or a 1541-bp fragment that includes no promoter. The *wip38,39* locus was amplified from a Wip4 template as either a 1287-bp fragment that includes 258-bp of promoter sequence or a 965-bp fragment that encodes no promoter. Resulting *bcp25,26* and *wip38,39* products were cloned into the *Sma*I sites of pASD2 or pWH1520. Promoter-bearing clones are referred to in the text with the prefix “P-“. The PlyG binding domain (PlyG^BD^) was amplified by PCR from a Wβ phage template with indicated primers and cloned in-frame with the 3′ end of *gfpmut2* in plasmid pBAD24 as described previously [46].

### Bacterial manipulations and growth conditions

Bacterial strains were grown in Luria broth (LB), brain-heart infusion broth (BHI), or Leighton-Doi broth [89] according to standard protocols; plates were made by adding Bacto^TM^ agar to a final concentration of 1.6%. Soil-extract and cellulose medium were prepared as described [90], [91]. Worm-extract plates were prepared as follows: 150 g of *Eisenia fetida* earthworms (New York Worms, Long Island, NY) were flash frozen, thawed, suspended in 1 liter of dH_2_0 and autoclaved. Agar (1.6%) was then added and the solution was re-autoclaved, cooled to 55°C, and poured into 150 mm wide plastic Petri dishes. When necessary, antibiotics were added to liquid or solid media at the following concentrations: tetracycline, 10 µg ml^−1^; kanamycin, 50 µg ml^−1^; spectinomycin, 250 µg ml^−1^, and ampicillin 100 µg ml^−1^. Activity of the P^BAD^ promoter in pBAD24 was induced during growth by the addition of L-arabinose to a final concentration of 0.2%. All *Bacillus* strains were grown at 30°C unless otherwise indicated. Aside from the production of *B. anthracis* mutants, all strains bearing pASD2 were maintained at 30°C to support its freely replicating, plasmidial form. Bacterial doubling times were determined using a standard laboratory technique.

Plasmids were introduced into electrocompetant *B. anthracis* using 0.4-cm gap cuvettes (BioRad) and conditions of 25 µF, 400Ω, and 2.5 kV in a GenePulser electroporation apparatus (Bio-Rad, Inc.). Plasmid pASD2 was used to insertionally inactivate *B. anthracis* ΔSterne loci according to a previously described method [87]. The general method used to construct *Bacillus* lysogens (unless otherwise stated) required mid-exponential phase cultures (grown in 5 ml BHI at 30°C with aeration at 150 rpm in 50 ml Falcon^TM^ tubes) to be infected with 1×10^8^ phage particles for 30 minutes, washed, and plated (undiluted and three 100-fold serial dilutions thereof) on BHI agar for 16 hours at 30°C. Representative colonies were subcultured on BHI agar and screened by PCR with phage-specific primers (including those in Table S7) to confirm lysogeny. In the standard 1 day infection protocols, frequencies of lysogeny ranged from 0.01 to 0.3. For Bcp1, ΔSterne lysogens were only obtained after infection with 5×10^3^ phage particles and incubation in the dark without aeration for 1 month. After the 1 month infection, all resulting colonies were lysogenic and assembled into biofilms. Phage curing was induced using a previously described heat treatment method [92]. Spontaneous phage curing was analyzed by plating overnight BHI cultures on BHI agar and screening 1×10^4^ resulting colonies for the rare colony morphology associated with loss of the indicated prophage. Putative cured derivatives were examined by PCR with phage-specific primers to confirm prophage loss.

### Phage identification and amplification

Environmental samples were prepared, according to standard protocols, from soil, salt and fresh water, herbivore feces, bat guano, landfill, silage, marine sludge, phylloplane washings, arthropod guts, and other sources. For solid materials, 1–5 g was added to 5–10 ml phosphate-buffered saline (PBS) and gently agitated at 4°C for 16 h. After centrifugation, phage-containing supernatants were recovered, passed through 0.2 µM filters, and 5 ml of filtrate was added to 5 ml of late-exponential phase ΔSterne. For Bcp1 identification, strain *B. cereus* T used instead. After overnight incubation at 30°C with aeration, supernatants were recovered, filtered, and used again to infect late-exponential phase bacterial cultures. After overnight incubation at 30°C with aeration, sterile supernatants were recovered, plated (undiluted and three serial 100-fold dilutions thereof) on fresh bacterial lawns on BHI plates, and incubated overnight at 30°C. Resulting phage plaques were recovered, plaque-purified, and, ultimately, used to infect 5 ml exponential phase bacterial cultures. Every two days, phage supernatants were collected and used to infect larger culture volumes.

Earthworms that yielded *B.anthracis*-active phage for this study were isolated as either free phages or induced prophages from forest leaf litter in Stroudsburg, PA (Wip1 and Wip4) and a compost heap in Southold, New York (Wip2 and Wip5). Other phages and their sources are as follows: Frp1 and Frp2, fern rhizosphere from Stroudsburg, PA; Htp1, a human tonsil from New York, NY: Bcp1, landfill soil in Port Washington, NY; and Slp1, from commercial potting soil (Miracle-Gro^®^). The Wβ phage was originally obtained as a lyosgenic phage of ATCC 11950 and is described elsewhere [46]. Total genomic DNA was obtained from each phage and subjected to agarose gel electrophoresis to confirm that the genome sizes were similar to that previously described [54] for members of the family *Tectiviridae* (∼16 kb), *Siphoviridae* (∼45 kb) and *Myoviridae* (∼140 kb). Partial genomic sequences ranging in size from one to five kilobases were then determined for each phage using a previously described metagenomic method [93]. Portions of the Wip1, Wip2, Wip4, Frp2, Htp1, and Bcp1 sequences are available in GenBank under accession numbers GQ214700, GQ214701, GQ221690, GQ214703, GQ214702, and EU930824, respectively. In addition to the sequence analysis, the distinct nature of each phage was confirmed by M13 fingerprinting analysis [94] Protein sequences for the recombination repair protein of phage Bcp1 and site-specific recombinase of phage Wip4 are have been submitted to GenBank under the names Bcp90 (GQ338829) and Wip21 (GQ338829), respectively.

### Spontaneous phage shedding in culture

Five ml overnight BHI cultures were washed twice with PBS, resuspended in 5 ml of PBS and used to inoculate 5 ml of either defibrinated sheep blood (Cleveland Scientific, Inc.), BHI, or LD medium at a dilution of 1∶100. After 24 hours of growth at 30°C with aeration, cultures were pelleted by centrifugation and the supernatant was recovered and filtered with 0.2 µM membranes. Sterile supernatants were titered on *B. anthracis* ΔSterne and resulting plaques were enumerated. Infections during co-culture are described in Table S5.

### Fluorescence studies

The GFP-PlyG^BD^ fusion protein was expressed and purified as described [46]. Alexa Fluor^®^-labeled wheat germ agglutinin (AF^WGA^) was obtained from Molecular Probes (Eugene, OR) and used according to the manufacturer's protocol. Biofilm samples were labeled with a GFP-PlyG^BD^ solution (1 mg ml^−1^) in PBS for 2 minutes at room temperature, rinsed with PBS, and visualized by microscopy. For the analysis of exponential phase bacteria, strains were grown for three hours at 30°C before 1 ml aliquots were removed, washed with PBS, and labeled with either GFP-PlyG^BD^ (1 mg ml^−1^ in PBS) or AF^WGA^. Samples were labeled for 2 minutes at room temperature, washed in PBS and then visualized by microscopy or subjected to an endpoint fluorescence analysis using a SpectraMax^®^ M5 microplate reader (Molecular Devices, Inc.). Relative fluorescence units (RFUs) are arbitrary values determined in black 96-well plates using excitation and emission wavelengths of 485 nm and 538 nm, respectively. GFP-PlyG^BD^ binds *B. anthracis*-specific neutral polysaccharide and WGA^AF^ is a carbohydrate-binding protein that recognizes N-acetylglucosaminyl sugars.

### Microscopy

Phase-contrast and fluorescence microscopy were performed with an Eclipse E400 microscope (Nikon) using the QCapture Pro^®^ version 5.1 imaging software. The TEM and SEM analyses were performed at The Rockefeller University Bio-Imaging Resource Center essentially as described [95], [96], [97]. Images were assembled using Adobe Photoshop 10.0.

### Sporulation studies

To assess the sporulation phenotype, overnight cultures were initially grown overnight in BHI supplemented with 0.2% glucose. The cultures were then washed twice, resuspended in LD, and used to inoculate 7.5 ml LD cultures at a 1∶100 dilution (in 50 ml Falcon^TM^ tubes). The cultures were incubated with indicated temperature and aeration conditions and at the indicated time points, aliquots were removed and either plated on BHI agar (for total viability) or heated for 15 min at 95°C, cooled for 5 min at 4°C, and plated for viability on BHI agar (for heat-resistant spore counts). The sporulation defects described in Table 2 were each separately confirmed using an assay for chloroform resistance [98]. For the analysis of sporulation in biofilms, culture tubes were vortexed to resolve all aggregated material prior to the heat resistance assay. For the analysis of spore ultrastructure, vegetative *B. anthracis* cells were first induced to sporulate by growth at 30°C for 4 days on either LD agar (for TEM) or cellulose agar (for SEM) in the dark. Spores were then purified to homogeneity using a water-washing method [98] and stored in dH_2_0 at 4°C in the dark.

### Construction and analysis of Bcp1 and Wip4 expression libraries

Phage libraries were generated essentially as described [99] with the exception that *Tsp509*I digested fragments in the 1.0–3.0 kb size range were cloned into pASD2. Complex plasmid pools of >1×10^4^ distinct clones were electroporated into *B. anthracis* ΔSterne. Spc- and kan-resistant clones were obtained at 30°C on BHI plates and replica plated to LD sporulation agar (with antibiotics) on glass plates. The glass plates were incubated for two days at 37°C, exposed to chloroform vapors for 30 min, and incubated again for 2 days at 37°C. Chloroform kills everything except spores, thus Spo^−^ colonies cannot re-grow after exposure and are easily distinguished from the majority background of Spo^+^ colonies. Colonies from the master plates, corresponding to dead Spo^−^ colonies on glass plates, were recovered and plasmid was prepared and sequenced. Five and seven clones were eventually identified from the Bcp1 and Wip4 libraries, respectively, with sporulation defects specifically assigned to plasmid insert expression. While insert sizes varied, each encoded either *bcp25,26* or *wip38,39*. The DNA sequences of *bcp25,26* and *wip38,39* are submitted to GenBank.

### Construction and analysis of the ΔSterne promoter-probe library

The chromosomal DNA of ΔSterne was isolated using Qiagen Genomic-tips according to the manufacturer's protocol. After partial digestion with *Tsp*509I, fragments in the 1- to 3-kb range were gel purified using the QIAquick gel purification system (Qiagen, Inc.) and ligated into the *Eco*RI site of dephosphorylated pASD2::*gfp*. The mix was transformed into *E. coli* XL10-Gold (Stratagene, Inc.) and ∼10,000 colonies were scraped up and used for plasmid purification with the Qiagen Midi kit. The library was then electroporated into *B. anthracis* ΔSterne/Wip4 and plated on worm-extract agar containing kanamycin and spectinomycin. After two days at 30°C the library consisting of ∼5000 clones was screened for fluorescence using a hand-held fluorescent lamp. Fifty clones displaying various fluorescence intensities were identified and subcultured to BHI agar for rescreening. Only one clone was then observed to not be fluorescent on BHI (but was on worm agar). Plasmid DNA was then recovered and ultimately used to transform ΔSterne. In the ΔSterne background, fluorescence was not observed on either worm or BHI agar. Sequence analysis revealed a 581-bp insert encoding both the 5′ end of *BA3436* as 356-bp of its upstream promoter region.

### Isolation and analysis of environmental *Bacillus* strains

The environmental isolates used in this study and the sources from which they were identified are described in Table S1. After recovery from the environment, samples were suspended in PBS to create a slurry, vortexed for 1 minute and plated (undiluted and three 100-fold serial dilutions thereof) on BHI agar. After overnight incubation at 30°C, the plates were screened for colony morphologies common to either *B. anthracis* (flat and matte) or *B. cereus* (shiny, gray-white). Colonies of interest were then subjected to the phenotypic and genotypic analyses shown in Table S1. A strain was considered *B. cereus* if it was β-hemolytic, fosfomycin-sensitive, and PCR-positive using primers directed against *plcR*, *BC5101*, *BC5449*, and *BC0442* of ATCC 14579. A strain was considered *B. anthracis* if it was not hemolytic, resistant to fosfomycin, and was PCR-positive with primers for virulence plasmid loci (*ggt*, *lef*, *cya*, and *pagA*), each of the chromosomal prophage (*BA4067*, *BA5353*, *BA0443*, and *BA3760*), and the marker Ceb-Bams 30 [100]. Surface binding to GFP-PlyG^BD^ and biofilm formation was assessed as described here. Prophages from the indicated strains in Table S1 were either spontaneously released into culture (Htp1) or induced by growth in BHI cultures supplemented with 150 µg ml^−1^ of fosfomycin [46]. Plaque-forming units were identified on a ΔSterne reporter strain. Strain RS1255 was isolated from a human tonsil that was acquired as part of an Internal Review Board (IRB)-approved protocol.

### RNA isolation and RT-PCR

RNA was extracted using RNeasy mini kits according to the manufacturer's protocol with the exception that the PlyG lysin [99] was added during lysis to improve RNA yield. An on-column DNase digest was performed using the RNase-free DNase set (Qiagen), followed by a second treatment with RNase-free DNase (Ambion, Inc.). 0.5 µg of RNA was then subject to a first strand cDNA synthesis using the Superscript III First Strand Synthesis Kit (Invitrogen, Inc.). The cDNA was analyzed with primer pairs indicated in Table S7.

### Biofilm formation and analysis

The *B. anthracis* biofilms were established from overnight 5 ml BHI cultures (with 0.2% glucose) that were washed twice in PBS, resuspended in 5 ml of PBS, and diluted 1∶1000 into 10 ml of BHI. After incubation for 3 months at room temperature in the dark without aeration, the cultures were photographed (Nikon CoolPix 5400) and processed for analysis. First, small biofilm sections were removed with a 200 µl pipet-tip, stained with GFP-PlyG^BD^ on a glass microscope slide, and analyzed by microscopy. Next, the liquid phase was carefully removed and biofilms were recovered into PBS and vortexed to resolve aggregated material. The samples were then plated for viability both before and after incubation at 95°C for 15 minutes.

The formation of biofilms by *B. cereus* RS1045 and its phage-cured derivative was examined using a different method. Five ml cultures were established in BHI containing 0.2% glucose and incubated overnight at 30°C with aeration. The next day, cultures were washed twice, resuspended in 5 ml LD medium, and used to inoculate, at a dilution of 1∶1000, 3 ml LD cultures in 12-well multi-well plates (non-treated polystyrene, BD Falcon^TM^). Sterile 22 mm^2^ glass coverslips (Fisherbrand) were then added to each well and incubated for three weeks without aeration in the dark. The coverslips were removed, washed gently with PBS, stained with crystal violet, and examined by microscopy.

### Soil microcosms

For the preparation of soil microcosms, 200 g of potting soil (Miracle-Gro^®^) was first added to 1 liter of dH_2_0 and autoclaved twice. Ten ml aliquots of the resulting slurry were transferred to 50 ml Falcon™ tubes and the indicated bacterial strains (5×10^8^ stationary-phase cells suspended in 0.3 ml dH_2_0) were added. All strains used here, including the lysogens, were transformed with pASD2 to facilitate recovery. The inoculated soil columns were then incubated at room temperature in the dark without aeration. At the indicated time points, 1 ml samples were removed, vortexed for 2 minutes and plated on BHI with antibiotics for viability both before and after heat-treatment at 95°C for 15 minutes. For the analysis of phage shedding, vortexed samples were also pelleted by centrifugation and supernatants were removed, filtered with 0.2 µM membranes, and titered on ΔSterne to determine PFUs ml^−1^ of culture.

For the analysis of phage infection in co-cultures, soil microcosms were prepared and inoculated with 5×10^8^ of the indicated *B. anthracis* lysogens (phage “donor” strains). None of the donor strains were antibiotic resistant. For some microcosms, 1×10^8^ Wip1 phages were added in lieu of donors. After 24 hours, 5×10^8^ each of the *B. anthracis* strains Sterne/pASD2 or ΔSterne/pASD2 (phage “recipient” strains) were added. The co-inoculated microcosms were incubated at room temperature in the dark without aeration. At the indicated times, samples were pelleted, resuspended in 5 ml PBS, and plated on BHI agar with antibiotics. Where indicated, samples were plated both before and after heat-treatment at 95°C for 15 minutes. Resulting colonies were subcultured and analyzed by PCR with phage-specific primers to evaluate infection with the indicated phages. Free phages in soil aliquots were determined by titering sterile-filtered supernatants on ΔSterne to determine PFUs ml^−1^ of culture.

### Earthworm microcosms

For earthworm colonizations, animals were washed twice in dH_2_0 and individually transferred using sterile forceps into separate 50 ml tubes containing ∼20 g potting soil (Miracle-Gro^®^). The worms were incubated at 24°C for 2 days, before 5×10^9^ stationary phase bacteria were added as suspensions in 5 ml dH_2_0. All strains used here, including the lysogens, were transformed with pASD2 to facilitate recovery. After 2 weeks, the worms were recovered from infected microcosms, washed twice in dH_2_0, and added to fresh soil. The worms were subsequently washed and placed in new soil every two weeks for 6 months. Ten separate microcosms were established for each strain. At indicated time points, worms were recovered, washed in dH_2_0, and their intestinal contents were forced out with sterile Pasteur pipettes. Their contents were suspended in 1 ml PBS, vortexed, and plated for viability on BHI with antibiotics both before and after heat-treatment at 95°C for 15 minutes.

For the analysis of phage infection during co-culture, earthworm microcosms were established and inoculated with *B. anthracis* lysogens (donors). None of the donor strains were antibiotic resistant. After 2 weeks, the worms were recovered, washed twice with dH_2_0, and returned to fresh soil. After one week, the worms were again washed and cycled through fresh soil for another week. Worms were then introduced into fresh soil and inoculated with either of the phage recipient *B. anthracis* strains ΔSterne/pASD2 and Sterne/pASD2 or 1×10^8^ Wip1 phages. After one week, the worms were then subjected to two week cycles of recovery, washing and re-introduction into fresh soil for a period of 6 months. At the indicated times, worms were recovered and the intestinal contents were removed and examined as above.

## Supporting Information

Table S1 (0.23 MB DOC)

Table S2 (0.23 MB DOC)

Table S3 (0.23 MB DOC)

Table S4 (0.23 MB DOC)

Table S5 (0.23 MB DOC)

Table S6 (0.22 MB DOC)

Table S7 (0.23 MB DOC)

Figure S1 The appearance of asporogenous (Spo^−^) *B. anthracis* lysogens. (A) A Spo^−^ Wip4 lysogen (indicated by arrow) appearing in field of Spo^+^ non-lysogens. Here, *B. anthracis* strain ΔSterne was infected with Wip4 (MOI of 0.01) for 16 hours in BHI liquid culture and plated for 24 hours on soil-extract agar. The indicated colony is ∼1–2 mm in diameter. (B) A Spo^−^ Frp1 lysogen (indicated by arrow) in field of Spo^+^ non-lysogens. Indicated colony is 4 mm in diameter. (C) The appearance of Spo^−^ derivatives of ΔSterne in Wip4-infected cultures. Here, mid-log phase liquid BHI cultures were infected with a range of phages concentrations (MOIs) for 3 hours, washed and plated for 16 hours on BHI. Resulting colonies were screened by PCR with phage-specific primers to identify lysogens. All lysogens corresponded to Spo^−^ colonies. Numbers are mean averages (n = 10) of Spo^−^ lysogens appearing in each condition and the error bars are standard deviations.
(0.99 MB TIF)

Figure S2 Bcp1 adsorbs to *B. anthracis* ΔSterne. Bacteria were infected with Bcp1 at an MOI of 1 (A) or 50 (B) for 15 minutes at 37°C, washed twice with PBS, fixed, and analyzed by thin-section electron microscopy. Scale bars are 50 nm. Arrows indicate phage heads that are either free (A) or full (B) of the Bcp1 genome. The absence of DNA in the phage head suggests that the genome translocated into ΔSterne.
(1.77 MB TIF)

Figure S3 Biofilms formed by *B. anthracis* and environmental *B. cereus* strains. Either settled material (for ΔSterne) or biofilms (for ΔSterne/Wip1, RS423, and RS421) formed at the liquid-air interface of 3 month-old BHI cultures grown without aeration at 24°C were recovered, labeled with GFP-PlyG^BD^, and examined by phase-constrast and fluorescence microscopy at 200X and 2000X magnifications. Exposure times are indicated for fluorescence images. (A) The biofilms of ΔSterne/Wip1 consist of a matrix enriched with the *B. anthracis* exopolysaccharide (the binding target of GFP-PlyG^BD^). Three distinct regions are observed in 2000X images, including spore/vegetative mixtures, vegetative-enriched, and spore-enriched zones from left to right. (B) Settled material in 3 month ΔSterne cultures consists predominantly of cellular debris that does not bind well to GFP-PlyG^BD^. ΔSterne alone does not form biolfims, thus only the settled material was analyzed. (C) Biofilms formed by RS423, a *B. cereus s.l.* strain from the worm gut, are in a GFP-PlyG^BD^-labeled matrix. (D) Biofilms formed by RS421, a *B. cereus s.l.* strain from the worm gut, are in a GFP-PlyG^BD^-labeled matrix.
(8.21 MB TIF)

Figure S4 Amino acid sequence alignment of known and putative bacterial sigma factors. Identical residues are highlighted by black backgrounds. Conserved amino acid changes are highlighted by gray backgrounds. SigF is the *B. anthracis* sigma factor, σ^F^, encoded by *BA4294*. The alignment was generated by ClustalW and displayed using the BOXSHADE program.
(0.03 MB DOC)

Figure S5 RT-PCR analysis of *bcp25,26* and *wip38,39* mRNA. We extracted mRNA from ΔSterne derivatives grown for 3 hours at 37°C in LD (A–D) or BHI (E) medium. The mRNA samples incubated either with (+) or without (−) reverse transcriptase (RT). Resulting cDNA was analyzed with primers listed to the left of each panel (sequences are in Table S7). Lane M is the 1 Kb Plus DNA ladder (New England Biolabs). Sizes in base pairs are at the right of each gel. (A) The ΔSterne/Bcp1 lyosgen. Amplifications were performed with primers bcp25-1,2 (lanes 1, 3 and 5) or bcp26-1,2 (lanes 2, 4, and 6) using either genomic DNA (lanes 3 and 4) or RT-treated (lanes 1 and 2) and untreated (lanes 5 and 6) mRNA. As controls, primers for *sap* (*BA0885*, a locus expressed during vegetative growth) and *sigF* (*BA4294*, a locus expressed only during sporulation) were used. (B) The ΔSterne/Wip4 lyosgen. Amplifications were performed with primers wip38-1,2 (lanes 1 and 3) or wip39-1,2 (lanes 2 and 4) using RT-treated (lanes 1 and 2) and untreated (lanes 5 and 6) mRNA. The wip38-1,2 primers span the intergenic region of *wip38* and *wip39*. (C) Analysis of ΔSterne/pASD2::P-*wip38,39* (Lanes 1 and 3) or ΔSterne/pASD2:: *wip38,39* ^PROMOTERLESS^ (lanes 2 and 4). The RT-treated samples were amplified with primers wip38-3,4 (lanes 1 and 2) or wip39-3,4 (lanes 3 and 4). The wip38-3,4 primers span the intergenic region of *wip38* and *wip39*. (D) Analysis of ΔSterne/pASD2::P-*bcp25,26* (Lanes 1, 3, and 5) or ΔSterne/pASD2::*bcp25,26* ^PROMOTERLESS^ (lanes 2, 4, 6). Amplifications were performed on RT-treated samples using primers bcp25-4,5 (lanes 1 and 2), bcp26-4,5 (lanes 3 and 4), or bcp25-3,bcp26-3 which span the *bcp25-bcp26* intergenic region (lanes 5 and 6). (E) Analysis ΔSterne lysogens during vegetative growth in BHI. Amplifications were performed on RT-treated samples from ΔSterne/Bcp1 (with bcp25-1,2 and bcp26-1,2 primers) and ΔSterne/Wip4 (with wip38-1,2 and wip39-1,2 primers).
(4.50 MB TIF)

Figure S6 Expanded analysis of survival in the soil and earthworm. Survival at indicated times after inoculation (solid lines) is shown as CFUs per gram of recovered soil or worm guts. Similarly, shedding of free phages (dashed lines) is shown as PFUs extracted per gram of soil or worm guts. Data is shown for *B. anthracis* viability (vegetative cells and spores; squares), spores alone (diamonds), and free phages (triangles). Values are reported as mean averages (n = 5) and error bars are standard deviations. (A) Soil survival for ΔSterne/pASD2 lysogens. (B) Earthworm gut survival for ΔSterne/pASD2 lysogens. (C) Survival of environmental *B.cereus* strain RS1045 and its phage-cured derivative (RS1045^CURED^) in the soil and earthworm gut. (D) Survival of environmental *B. anthracis* strain RS1046 and its phage-cured derivative (RS1046^CURED^) in the soil and earthworm gut. (E) Infection of *B. anthracis* during co-culture with lysogens in soil microcosms. Strains ΔSterne/pASD2 and Sterne/pASD2 were either inoculated alone or with *B. cereus* RS1045 or *B. anthracis* RS1046. At the indicated time points, ΔSterne/pASD2 and Sterne/pASD2 were selectively recovered and scored by PCR for infection with either Wip4 (the phage shed by RS1045) or Wip5 (the phage shed by RS1046). Survival of ΔSterne/pASD2 and Sterne/pASD2 inoculated alone (squares) and their derivatives that have become stably infected with Wip4 (closed circles) or Wip5 (open circles) are shown.
(2.16 MB TIF)

Figure S7 Microscopic analysis of *B. anthracis* strains recovered from soil microcosms. Culture aliquots of indicated strains were removed at 3 months, labeled with GFP-PlyG^BD^, and analyzed. Phase-contrast and corresponding fluorescence images are shown at 200X and 2000X magnification. The exposure time for each image was 0.3 seconds.
(9.51 MB TIF)

Figure S8 The shedding of bacteriophage by *B. anthracis* and its lysogens. *B. anthracis* strain ΔSterne, its indicated lysogens, and the environmental *B. anthracis* strain RS1615 were examined. Numbers are mean averages (n = 5) of PFUs shed into the media during the culture of each stain and the error bars are standard deviations.
(1.09 MB TIF)
