# Supplementary material for: The Secret Life of the Anthrax Agent Bacillus anthracis: Bacteriophage-Mediated Ecological Adaptations
Source: PLoS One. 2009 Aug 12;4(8):e6532. doi: 10.1371/journal.pone.0006532 (PMC2716549; doi:10.1371/journal.pone.0006532)
Supplement: Table S1 — (0.23 MB DOC) [file pone.0006532.s001.doc]

**Table S1.** Phenotypic and genotypic analysis of *B. anthracis* and *B. cereus* strains.

| **Strain** | **Source** | **Hem** | **Fos^R^** | **Ba^CHR^** | **Ba^VIR^** | **PlyG^BD^** | **Biofilm** | **φ** | **Group** |
| --- | --- | --- | --- | --- | --- | --- | --- | --- | --- |
| ***B.anthracis*** |  |  |  |  |  |  |  |  |  |
| **ΔSterne** | Lab strain | - | + | + | - | + | - | no |  |
| **Sterne** | Lab strain | - | + | + | + | + | + | φ20* |  |
| ***B. cereus*** |  |  |  |  |  |  |  |  |  |
| **T** | Lab strain | + | - | - | - | - | - | no |  |
| **ATCC 4342** | Lab strain | + | - | - | - | + | - | no |  |
| **ATCC 25621** | Cow dung | - | - | - | - | + | + | φ415 |  |
| **Environ. strains** |  |  |  |  |  |  |  |  |  |
| **RS1615** | Soil | - | + | + | + | + | + | φ1615 | *anthracis* |
| **RS1046** | Worm gut | - | + | + | - | + | + | Wip5 | *anthracis* |
| **RS1255** | Human tonsil | + | - | - | - | - | + | Htp1 | *cereus* |
| **RS1045** | Worm gut | + | + | - | - | + | + | Wip4 | *cereus* |
| **RS1557** | Rhizosphere | + | + | - | - | - | + | Frp2 | *cereus* |
| **RS1047** | Worm gut | + | + | - | - | - | + | φ1047 | *cereus* |
| **RS421** | Worm gut | + | n.d. | - | - | - | + | φ421 | *cereus* |
| **RS423** | Worm gut | - | n.d. | - | - | - | + | φ423 | *cereus* |

Environmental strains identified in this study were compared to known *B. anthracis* and *B. cereus* isolates in the following phenotypic and genotypic analyses: Hem, β-hemolysis on blood agar plates; Fos^R^, growth in culture supplemented with 150 mg fosfomycin ml^-1^; Ba^CHR^, PCR-positive for *B. anthracis*-specific chromosomal loci at the four native, non-inducible prophage sequences and the diagnostic marker Ceb-Bams 30; Ba^VIR^, PCR-positive for the *B. anthracis* virulence plasmid loci; PlyG^BD^, binding to GFP-PlyG^BD^; Biofilm, formation of multicellular structures at the liquid-air interface of 10 ml LD cultures incubated for 2 months at room temperature; φ, the name given to an inducible *B. anthracis*-infective phage identified in that strain (“no” indicates that no phage was observed); Group, indicates a preliminary taxonomic assignment based on the findings in this work. *, denotes the fact that φ20 has been described in previous work. “n.d.” indicates that the experiment was not done.

|  |  |
| --- | --- |
|  |  |
|  |  |
|  |  |
|  |  |
|  |  |
|  |  |

Bcp1 adsorption characteristics.

|  |  |  |
| --- | --- | --- |
|  |  |  |
|  |  |  |
|  |  |  |
|  |  |  |

|  |  |  |  |  |
| --- | --- | --- | --- | --- |
|  |  |  |  |  |
|  |  |  |  |  |
|  |  |  |  |  |
|  |  |  |  |  |
|  |  |  |  |  |
|  |  |  |  |  |
|  |  |  |  |  |

|  |  |  |  |
| --- | --- | --- | --- |
|  |  |  |  |
|  |  |  |  |
|  |  |  |  |
|  |  |  |  |

Bacterial strains and plasmids used in this study.

|  |  |  |
| --- | --- | --- |
|  |  |  |
|  |  |  |
|  |  |  |
|  |  |  |
|  |  |  |
|  |  |  |
|  |  |  |
|  |  |  |
|  |  |  |
|  |  |  |
|  |  |  |
|  |  |  |
|  |  |  |
|  |  |  |
|  |  |  |
|  |  |  |
|  |  |  |
|  |  |  |
|  |  |  |
|  |  |  |
|  |  |  |
|  |  |  |
|  |  |  |
|  |  |  |
|  |  |  |
|  |  |  |
|  |  |  |
|  |  |  |
|  |  |  |
|  |  |  |
|  |  |  |
|  |  |  |
|  |  |  |
|  |  |  |

Select primers used in this study.

|  |  |  |
| --- | --- | --- |
|  |  |  |
|  |  |  |
|  |  |  |
|  |  |  |
|  |  |  |
|  |  |  |
|  |  |  |
|  |  |  |
|  |  |  |
|  |  |  |
|  |  |  |
|  |  |  |
|  |  |  |
|  |  |  |
|  |  |  |
|  |  |  |
|  |  |  |
|  |  |  |
|  |  |  |
|  |  |  |
|  |  |  |
|  |  |  |
|  |  |  |
|  |  |  |
|  |  |  |
|  |  |  |
|  |  |  |
|  |  |  |
|  |  |  |
|  |  |  |
|  |  |  |
|  |  |  |
|  |  |  |
|  |  |  |
|  |  |  |
|  |  |  |
|  |  |  |
|  |  |  |
|  |  |  |
|  |  |  |
|  |  |  |
|  |  |  |
|  |  |  |
|  |  |  |
|  |  |  |
|  |  |  |
|  |  |  |
|  |  |  |
|  |  |  |
|  |  |  |
|  |  |  |
|  |  |  |
|  |  |  |
|  |  |  |
|  |  |  |
|  |  |  |
|  |  |  |
|  |  |  |
|  |  |  |
|  |  |  |
|  |  |  |
|  |  |  |
|  |  |  |
|  |  |  |
|  |  |  |
|  |  |  |
|  |  |  |
|  |  |  |
