# Supplementary material for: The Secret Life of the Anthrax Agent Bacillus anthracis: Bacteriophage-Mediated Ecological Adaptations
Source: PLoS One. 2009 Aug 12;4(8):e6532. doi: 10.1371/journal.pone.0006532 (PMC2716549; doi:10.1371/journal.pone.0006532)
Supplement: Table S4 — (0.23 MB DOC) [file pone.0006532.s004.doc]

strains.

|  |  |  |  |  |  |  |  |  |  |
| --- | --- | --- | --- | --- | --- | --- | --- | --- | --- |
|  |  |  |  |  |  |  |  |  |  |
|  |  |  |  |  |  |  |  |  |  |
|  |  |  |  |  |  |  |  |  |  |
|  |  |  |  |  |  |  |  |  |  |
|  |  |  |  |  |  |  |  |  |  |
|  |  |  |  |  |  |  |  |  |  |
|  |  |  |  |  |  |  |  |  |  |
|  |  |  |  |  |  |  |  |  |  |
|  |  |  |  |  |  |  |  |  |  |
|  |  |  |  |  |  |  |  |  |  |
|  |  |  |  |  |  |  |  |  |  |
|  |  |  |  |  |  |  |  |  |  |
|  |  |  |  |  |  |  |  |  |  |
|  |  |  |  |  |  |  |  |  |  |
|  |  |  |  |  |  |  |  |  |  |
|  |  |  |  |  |  |  |  |  |  |

|  |  |
| --- | --- |
|  |  |
|  |  |
|  |  |
|  |  |
|  |  |
|  |  |

Bcp1 adsorption characteristics.

|  |  |  |
| --- | --- | --- |
|  |  |  |
|  |  |  |
|  |  |  |
|  |  |  |

**Table S4.** Effect of lysogeny on the sporulation (Spo) phenotype of *B. anthracis* ΔSterne.

| **Infecting phage** | **Bacteria ml^-1^** | **Spo^-^ bacteria ml^-1^** | **Frequency of Spo^+^ lysogens (%)** | **Frequency of Spo^-^ lysogens (%)** |
| --- | --- | --- | --- | --- |
| **Wβ** | 1.0 ± 0.1 x 10^9^ | <4.0 x 10^5^ | 18.4 | <0.04 |
| **Wip2** | 7.7 ± 0.9 x 10^8^ | <4.0 x 10^5^ | 15.3 | <0.05 |
| **Wip4** | 8.7 ± 1.4 x 10^8^ | 3.2 ± 1.2 x10^8^ | <0.4 | 36.7 |
| **Wip5** | 5.7 ± 0.9 x 10^8^ | 2.5 ± 0.4 x10^8^ | <0.7 | 43.8 |
| **Frp1** | 6.7 ± 0.5 x 10^8^ | 1.6 ± 0.4 x10^8^ | <0.5 | 23.8 |
| **Frp1 (heat-killed)** | 6.2 ± 0.4 x 10^8^ | <4.0 x 10^5^ | <0.6 | <0.06 |
| **No phage** | 7.1 ± 0.9 x 10^8^ | <4.0 x 10^5^ | n.a. | n.a. |

Cultures were infected (MOI=0.5) for 30 minutes, washed, and plated at various dilutions on BHI agar. Resulting bacterial colonies were examined for colony morphology and enumerated. Large, opaque colonies are Spo^-^, while small, matte colonies are Spo^+^. Spo phenotypes were confirmed by microscopic analysis of colonies subcultured on LD sporulation agar. Stable lysogens were confirmed by PCR with phage-specific primers. As a control, heat-killed Frp1 samples were incubated at 65°C for 30 min prior to infection to destroy its infective capacity. The frequency of lysogenization among infected cells [(stable lysogens/total number of cells) X 100] was determined and is shown. Threshold levels for detecting Spo^+^ and Spo^-^ lysogens were 4 x10^5^ and 4 x10^6^ bacteria ml^-1^, respectively. Experiments were performed in triplicate. “n.a.” indicates that the experiment was not applicable here.

|  |  |  |  |
| --- | --- | --- | --- |
|  |  |  |  |
|  |  |  |  |
|  |  |  |  |
|  |  |  |  |

Bacterial strains and plasmids used in this study.

|  |  |  |
| --- | --- | --- |
|  |  |  |
|  |  |  |
|  |  |  |
|  |  |  |
|  |  |  |
|  |  |  |
|  |  |  |
|  |  |  |
|  |  |  |
|  |  |  |
|  |  |  |
|  |  |  |
|  |  |  |
|  |  |  |
|  |  |  |
|  |  |  |
|  |  |  |
|  |  |  |
|  |  |  |
|  |  |  |
|  |  |  |
|  |  |  |
|  |  |  |
|  |  |  |
|  |  |  |
|  |  |  |
|  |  |  |
|  |  |  |
|  |  |  |
|  |  |  |
|  |  |  |
|  |  |  |
|  |  |  |
|  |  |  |

Select primers used in this study.

|  |  |  |
| --- | --- | --- |
|  |  |  |
|  |  |  |
|  |  |  |
|  |  |  |
|  |  |  |
|  |  |  |
|  |  |  |
|  |  |  |
|  |  |  |
|  |  |  |
|  |  |  |
|  |  |  |
|  |  |  |
|  |  |  |
|  |  |  |
|  |  |  |
|  |  |  |
|  |  |  |
|  |  |  |
|  |  |  |
|  |  |  |
|  |  |  |
|  |  |  |
|  |  |  |
|  |  |  |
|  |  |  |
|  |  |  |
|  |  |  |
|  |  |  |
|  |  |  |
|  |  |  |
|  |  |  |
|  |  |  |
|  |  |  |
|  |  |  |
|  |  |  |
|  |  |  |
|  |  |  |
|  |  |  |
|  |  |  |
|  |  |  |
|  |  |  |
|  |  |  |
|  |  |  |
|  |  |  |
|  |  |  |
|  |  |  |
|  |  |  |
|  |  |  |
|  |  |  |
|  |  |  |
|  |  |  |
|  |  |  |
|  |  |  |
|  |  |  |
|  |  |  |
|  |  |  |
|  |  |  |
|  |  |  |
|  |  |  |
|  |  |  |
|  |  |  |
|  |  |  |
|  |  |  |
|  |  |  |
|  |  |  |
|  |  |  |
|  |  |  |
