# Supplementary material for: The Secret Life of the Anthrax Agent Bacillus anthracis: Bacteriophage-Mediated Ecological Adaptations
Source: PLoS One. 2009 Aug 12;4(8):e6532. doi: 10.1371/journal.pone.0006532 (PMC2716549; doi:10.1371/journal.pone.0006532)
Supplement: Table S5 — (0.23 MB DOC) [file pone.0006532.s005.doc]

strains.

|  |  |  |  |  |  |  |  |  |  |
| --- | --- | --- | --- | --- | --- | --- | --- | --- | --- |
|  |  |  |  |  |  |  |  |  |  |
|  |  |  |  |  |  |  |  |  |  |
|  |  |  |  |  |  |  |  |  |  |
|  |  |  |  |  |  |  |  |  |  |
|  |  |  |  |  |  |  |  |  |  |
|  |  |  |  |  |  |  |  |  |  |
|  |  |  |  |  |  |  |  |  |  |
|  |  |  |  |  |  |  |  |  |  |
|  |  |  |  |  |  |  |  |  |  |
|  |  |  |  |  |  |  |  |  |  |
|  |  |  |  |  |  |  |  |  |  |
|  |  |  |  |  |  |  |  |  |  |
|  |  |  |  |  |  |  |  |  |  |
|  |  |  |  |  |  |  |  |  |  |
|  |  |  |  |  |  |  |  |  |  |
|  |  |  |  |  |  |  |  |  |  |

|  |  |
| --- | --- |
|  |  |
|  |  |
|  |  |
|  |  |
|  |  |
|  |  |

Bcp1 adsorption characteristics.

|  |  |  |
| --- | --- | --- |
|  |  |  |
|  |  |  |
|  |  |  |
|  |  |  |

|  |  |  |  |  |
| --- | --- | --- | --- | --- |
|  |  |  |  |  |
|  |  |  |  |  |
|  |  |  |  |  |
|  |  |  |  |  |
|  |  |  |  |  |
|  |  |  |  |  |
|  |  |  |  |  |

**Table S5.** Frequency of *B. anthracis* derivatives infected with phages that are shed by lysogens during growth in different media types.

| **Phage donor (lysogen)** | **Sheep Blood** | **BHI** | **LD** |
| --- | --- | --- | --- |
| **ΔSterne/Wβ** | 0.027 | <1.5x10^-4^ | <7.6x10^-5^ |
| **ΔSterne/Wip1** | 0.016 | <1.6x10^-4^ | <5.0x10^-4^ |
| **ΔSterne/Bcp1** | <1.6x10^-4^ | 0.02 | 0.001 |
| **ΔSterne/Wip4** | <1.5x10^-4^ | 0.3 | 0.02 |

Overnight cultures of *B. anthracis* ΔSterne/pASD2 (kanamycin- and spectinomycin-resistant) were co-inoculated with indicated lysogens into one of three types of growth media and incubated for 2 days at 30°C with aeration. Culture aliquots and dilutions thereof were plated on BHI agar supplemented with antibiotics to select for ΔSterne/pASD2 survivors and against the phage donor strains. Infection of ΔSterne/pASD2 by shed phages was determined by PCR analysis with phage-specific primers of >200 ΔSterne/pASD2 colonies resulting from each condition. The reported frequency was determined based on the number of PCR-positive derivatives per ml of culture/the total number of ΔSterne/pASD2 bacteria per ml of culture. “<” indicates that stable infection, if any, was below the threshold of detection. These experiments were performed in duplicate and all values represent averages.

Bacterial strains and plasmids used in this study.

|  |  |  |
| --- | --- | --- |
|  |  |  |
|  |  |  |
|  |  |  |
|  |  |  |
|  |  |  |
|  |  |  |
|  |  |  |
|  |  |  |
|  |  |  |
|  |  |  |
|  |  |  |
|  |  |  |
|  |  |  |
|  |  |  |
|  |  |  |
|  |  |  |
|  |  |  |
|  |  |  |
|  |  |  |
|  |  |  |
|  |  |  |
|  |  |  |
|  |  |  |
|  |  |  |
|  |  |  |
|  |  |  |
|  |  |  |
|  |  |  |
|  |  |  |
|  |  |  |
|  |  |  |
|  |  |  |
|  |  |  |
|  |  |  |

Select primers used in this study.

|  |  |  |
| --- | --- | --- |
|  |  |  |
|  |  |  |
|  |  |  |
|  |  |  |
|  |  |  |
|  |  |  |
|  |  |  |
|  |  |  |
|  |  |  |
|  |  |  |
|  |  |  |
|  |  |  |
|  |  |  |
|  |  |  |
|  |  |  |
|  |  |  |
|  |  |  |
|  |  |  |
|  |  |  |
|  |  |  |
|  |  |  |
|  |  |  |
|  |  |  |
|  |  |  |
|  |  |  |
|  |  |  |
|  |  |  |
|  |  |  |
|  |  |  |
|  |  |  |
|  |  |  |
|  |  |  |
|  |  |  |
|  |  |  |
|  |  |  |
|  |  |  |
|  |  |  |
|  |  |  |
|  |  |  |
|  |  |  |
|  |  |  |
|  |  |  |
|  |  |  |
|  |  |  |
|  |  |  |
|  |  |  |
|  |  |  |
|  |  |  |
|  |  |  |
|  |  |  |
|  |  |  |
|  |  |  |
|  |  |  |
|  |  |  |
|  |  |  |
|  |  |  |
|  |  |  |
|  |  |  |
|  |  |  |
|  |  |  |
|  |  |  |
|  |  |  |
|  |  |  |
|  |  |  |
|  |  |  |
|  |  |  |
|  |  |  |
|  |  |  |
