# Supplementary material for: The Secret Life of the Anthrax Agent Bacillus anthracis: Bacteriophage-Mediated Ecological Adaptations
Source: PLoS One. 2009 Aug 12;4(8):e6532. doi: 10.1371/journal.pone.0006532 (PMC2716549; doi:10.1371/journal.pone.0006532)
Supplement: Table S6 — (0.22 MB DOC) [file pone.0006532.s006.doc]

strains.

|  |  |  |  |  |  |  |  |  |  |
| --- | --- | --- | --- | --- | --- | --- | --- | --- | --- |
|  |  |  |  |  |  |  |  |  |  |
|  |  |  |  |  |  |  |  |  |  |
|  |  |  |  |  |  |  |  |  |  |
|  |  |  |  |  |  |  |  |  |  |
|  |  |  |  |  |  |  |  |  |  |
|  |  |  |  |  |  |  |  |  |  |
|  |  |  |  |  |  |  |  |  |  |
|  |  |  |  |  |  |  |  |  |  |
|  |  |  |  |  |  |  |  |  |  |
|  |  |  |  |  |  |  |  |  |  |
|  |  |  |  |  |  |  |  |  |  |
|  |  |  |  |  |  |  |  |  |  |
|  |  |  |  |  |  |  |  |  |  |
|  |  |  |  |  |  |  |  |  |  |
|  |  |  |  |  |  |  |  |  |  |
|  |  |  |  |  |  |  |  |  |  |

|  |  |
| --- | --- |
|  |  |
|  |  |
|  |  |
|  |  |
|  |  |
|  |  |

Bcp1 adsorption characteristics.

|  |  |  |
| --- | --- | --- |
|  |  |  |
|  |  |  |
|  |  |  |
|  |  |  |

|  |  |  |  |  |
| --- | --- | --- | --- | --- |
|  |  |  |  |  |
|  |  |  |  |  |
|  |  |  |  |  |
|  |  |  |  |  |
|  |  |  |  |  |
|  |  |  |  |  |
|  |  |  |  |  |

|  |  |  |  |
| --- | --- | --- | --- |
|  |  |  |  |
|  |  |  |  |
|  |  |  |  |
|  |  |  |  |

**Table S6.** Bacterial strains and plasmids used in this study.

| **Strain or plasmid** | **Genotype and description^1^** | **Reference or source** |
| --- | --- | --- |
| ***B. anthracis*** |  |  |
| **Sterne** | pX01^+^ strain 34F2 | J. Lederberg |
| **ΔSterne** | Plasmid-cured 34F2 | J. Lederberg |
| **ΔSterne::Wip4 *ΔBA3443*** | *BA3443* insertional mutant | This study |
| **ΔSterne::Wip4 *ΔBA4109*** | *BA4109* insertional mutant | This study |
| **ΔSterne::Wip4* ΔBA0672*** | *BA0672* insertional mutant | This study |
| **ΔSterne::Wip4* ΔBA1295*** | *BA1295* insertional mutant | This study |
| ***B. cereus*** |  |  |
| **ATCC 14579** | Reference strain | ATCC |
| **ATCC 25621** | Cow dung isolate | ATCC |
| **T** | Laboratory strain | A.Aronson |
| **ATCC 11950** | Source of Wβ phage | [46] |
| **Plasmids** |  |  |
| **pBAD24** | Arabinose-inducible *E. coli* expression vector | [98] |
| **pBAD24::*gfp-plyG^BD^*** | Production of GFP-PlyG^BD^ | [46] |
| **pASD2** | Kan^R^ Spc^R^ Amp^R^ *E. coli*-*B. anthracis* shuttle vector | [82] |
| **pASD2::*gfp*** | *gfpmut2* vector | This study |
| **pASD2::P*^BA3443^*-*gfp*** | *BA3443* promoter-*gfpmut2* fusion vector | This study |
| **pASD2::P*^BA0672^*-*gfp*** | *BA0672* promoter-*gfpmut2* fusion vector | This study |
| **pASD2::P*^BA1295^*-*gfp*** | *BA1295* promoter-*gfpmut2* fusion vector | This study |
| **pASD2::P*^BA3436^*-*gfp*** | *BA3436* promoter-*gfpmut2* fusion vector | This study |
| **pASD2::*ΔBA3443*** | *BA3443* mutagenesis vector | This study |
| **pASD2::*ΔBA4109*** | *BA4109* mutagenesis vector | This study |
| **pASD2::*ΔBA0672*** | *BA0672* mutagenesis vector | This study |
| **pASD2::*ΔBA1295*** | *BA1295* mutagenesis vector | This study |
| **pASD2::*Δbcp25*** | *bcp25* mutagenesis vector | This study |
| **pASD2::*Δwip39*** | *wip39* mutagenesis vector | This study |
| **pASD2::P-*bcp25,26*** | Fragment bearing *bcp25,26* locus with 353-bp promoter | This study |
| **pASD2::P-*wip38,39*** | Fragment bearing *wip38,39* locus with 258-bp promoter | This study |
| **pASD2::*bcp25,26*** | Fragment bearing *bcp25,26* locus with no promoter | This study |
| **pASD2::*wip38,39*** | Fragment bearing *wip38,39* locus with no promoter | This study |
| **pWH1520** | Tc^R^ Amp^R^ *E. coli*-*Bacillus* shuttle vector | MoBiTec, Inc. |
| **pWH1520::*bcp25,26*** | Fragment bearing *bcp25,26* locus with no promoter | This study |
| **pWH1520::*wip38,39*** | Fragment bearing *wip38,39* locus with no promoter | This study |

^1^Kan, kanamycin; Spc,spectinomycin; Tc, tetracycline; Amp, ampicillin.

^2^American Type Culture Collection (Manassas, VA).

Select primers used in this study.

|  |  |  |
| --- | --- | --- |
|  |  |  |
|  |  |  |
|  |  |  |
|  |  |  |
|  |  |  |
|  |  |  |
|  |  |  |
|  |  |  |
|  |  |  |
|  |  |  |
|  |  |  |
|  |  |  |
|  |  |  |
|  |  |  |
|  |  |  |
|  |  |  |
|  |  |  |
|  |  |  |
|  |  |  |
|  |  |  |
|  |  |  |
|  |  |  |
|  |  |  |
|  |  |  |
|  |  |  |
|  |  |  |
|  |  |  |
|  |  |  |
|  |  |  |
|  |  |  |
|  |  |  |
|  |  |  |
|  |  |  |
|  |  |  |
|  |  |  |
|  |  |  |
|  |  |  |
|  |  |  |
|  |  |  |
|  |  |  |
|  |  |  |
|  |  |  |
|  |  |  |
|  |  |  |
|  |  |  |
|  |  |  |
|  |  |  |
|  |  |  |
|  |  |  |
|  |  |  |
|  |  |  |
|  |  |  |
|  |  |  |
|  |  |  |
|  |  |  |
|  |  |  |
|  |  |  |
|  |  |  |
|  |  |  |
|  |  |  |
|  |  |  |
|  |  |  |
|  |  |  |
|  |  |  |
|  |  |  |
|  |  |  |
|  |  |  |
|  |  |  |
