# Supplementary material for: The Secret Life of the Anthrax Agent Bacillus anthracis: Bacteriophage-Mediated Ecological Adaptations
Source: PLoS One. 2009 Aug 12;4(8):e6532. doi: 10.1371/journal.pone.0006532 (PMC2716549; doi:10.1371/journal.pone.0006532)
Supplement: Table S7 — (0.23 MB DOC) [file pone.0006532.s007.doc]

strains.

|  |  |  |  |  |  |  |  |  |  |
| --- | --- | --- | --- | --- | --- | --- | --- | --- | --- |
|  |  |  |  |  |  |  |  |  |  |
|  |  |  |  |  |  |  |  |  |  |
|  |  |  |  |  |  |  |  |  |  |
|  |  |  |  |  |  |  |  |  |  |
|  |  |  |  |  |  |  |  |  |  |
|  |  |  |  |  |  |  |  |  |  |
|  |  |  |  |  |  |  |  |  |  |
|  |  |  |  |  |  |  |  |  |  |
|  |  |  |  |  |  |  |  |  |  |
|  |  |  |  |  |  |  |  |  |  |
|  |  |  |  |  |  |  |  |  |  |
|  |  |  |  |  |  |  |  |  |  |
|  |  |  |  |  |  |  |  |  |  |
|  |  |  |  |  |  |  |  |  |  |
|  |  |  |  |  |  |  |  |  |  |
|  |  |  |  |  |  |  |  |  |  |

|  |  |
| --- | --- |
|  |  |
|  |  |
|  |  |
|  |  |
|  |  |
|  |  |

Bcp1 adsorption characteristics.

|  |  |  |
| --- | --- | --- |
|  |  |  |
|  |  |  |
|  |  |  |
|  |  |  |

|  |  |  |  |  |
| --- | --- | --- | --- | --- |
|  |  |  |  |  |
|  |  |  |  |  |
|  |  |  |  |  |
|  |  |  |  |  |
|  |  |  |  |  |
|  |  |  |  |  |
|  |  |  |  |  |

|  |  |  |  |
| --- | --- | --- | --- |
|  |  |  |  |
|  |  |  |  |
|  |  |  |  |
|  |  |  |  |

Bacterial strains and plasmids used in this study.

|  |  |  |
| --- | --- | --- |
|  |  |  |
|  |  |  |
|  |  |  |
|  |  |  |
|  |  |  |
|  |  |  |
|  |  |  |
|  |  |  |
|  |  |  |
|  |  |  |
|  |  |  |
|  |  |  |
|  |  |  |
|  |  |  |
|  |  |  |
|  |  |  |
|  |  |  |
|  |  |  |
|  |  |  |
|  |  |  |
|  |  |  |
|  |  |  |
|  |  |  |
|  |  |  |
|  |  |  |
|  |  |  |
|  |  |  |
|  |  |  |
|  |  |  |
|  |  |  |
|  |  |  |
|  |  |  |
|  |  |  |
|  |  |  |

**Table S7.** Select primers used in this study.

| **Name** | **Sequence** | **Function** |
| --- | --- | --- |
| **Wip1 up** | 5’-actggaaaacaccctgac-3’ | Confirm lysogeny |
| **Wip1 down** | 5’-gtggttcaaatgcggttct-3’ | Confirm lysogeny |
| **Wip2 up** | 5’-gcttctgcgttctgatttaatctg-3’ | Confirm lysogeny |
| **Wip2 down** | 5’-ggtcaaaagtatacattcgttgttaaagg-3’ | Confirm lysogeny |
| **Wip4 up** | 5’-cgaattacttttccgagtggcaatg-3’ | Confirm lysogeny |
| **Wip4 down** | 5’-ccccatcacttggcgctttc-3’ | Confirm lysogeny |
| **Wip5 up** | 5’-caaattctgttttatcagaccgcttctg-3’ | Confirm lysogeny |
| **Wip5 down** | 5’-ctccactcaatttcactatgaaaatcg-3’ | Confirm lysogeny |
| **Slp1 up** | 5’-gtagaaggtgtagcttatattgaagg-3’ | Confirm lysogeny |
| **Slp1 down** | 5’-ctattgtcaatggtggatcattgg-3’ | Confirm lysogeny |
| **Bcp1 up** | 5’-atgggttacatcgttgatatg-3’ | Confirm lysogeny |
| **Bcp1 down** | 5’-ttacttgaatgtgccccaag-3’ | Confirm lysogeny |
| **Wβ up** | 5’-atgagaattgcgctttacag-3’ | Confirm lysogeny |
| **Wβ down** | 5’-ttatacgacaatcccactgtt-3’ | Confirm lysogeny |
| **bcp25,26 pro up** | 5’-ctcccgggaattcgtagctaaatgcggttttagatta-3’ | Expression clone |
| **bcp25,26 pro down** | 5’-ctcccgggaatttaatagctgcttgctttttgctttcaaatg-3’ | Expression clone |
| **bcp25,26 up** | 5’-ctcccgggatgaataagaaaagggcaggtagcac-3’ | Expression clone |
| **bcp25,26 down** | 5’-ctcccgggttaaactcctcctagttctaatttaatt-3’ | Expression clone |
| **wip38,39 pro up** | 5’-ctgatatcaattatggcattactcatgaaatgtgatg-3’ | Expression clone |
| **wip38,39 pro down** | 5’-ctgatatcaattctactttctcctttagcaaatct-3’ | Expression clone |
| **wip38,39 up** | 5’-ctgatatcatggctaataataaaaacggggaac-3’ | Expression clone |
| **wip38,39 down** | 5’-ctgatatctcaatcttgaagacctccttctttacc-3’ | Expression clone |
| **BA3443 pro up** | 5’-ctgaattcaattggaaacggattgcgcaaatgcc-3’ | Promoter-*gfp* fusion |
| **BA3443 pro down** | 5’-ctgaattcaatttcttttacatttgttcgttcgtc-3’ | Promoter-*gfp* fusion |
| **BA0672 pro up** | 5’-ctgaattcaattgctccaaacgttgttgtaaaggttc-3’ | Promoter-*gfp* fusion |
| **BA0672 pro down** | 5’-ctgaattcaattggtgttgtagatacattttctttc-3’ | Promoter-*gfp* fusion |
| **BA1295 pro up** | 5’-ctgaattcaattgtgatatactcgtatgctaactatg-3’ | Promoter-*gfp* fusion |
| **BA1295 pro down** | 5’-ctgaattcaattctgataaatcaagaccgctatctacag-3’ | Promoter-*gfp* fusion |
| **BA3436 pro up** | 5’-ctgaattcaatttggatattgagcattttatataaag-3’ | Promoter-*gfp* fusion |
| **BA3436 pro down** | 5’-ctgaattcaattttcaatcggaatgtggactcc-3’ | Promoter-*gfp* fusion |
| **gfpmut2 up** | 5’-gatggtaccgaattctaagaaggagatatacatatgagtaaagg-3’ | Promoter-*gfp* fusion |
| **gfpmut2 down** | 5’-catgatatccttatttgtatagttcatccatgccatg-3’ | Promoter-*gfp* fusion |
| **BA3443KO1** | 5’-acaggtacccatgctgacgaacgaacaaatgta-3’ | mutagenesis |
| **BA3443KO2** | 5’-acaggtaccaatcggaagaggtttactagcactt-3’ | mutagenesis |
| **BA0672KO1** | 5’-acaggtaccatgagtgctccattagcgtacg-3’ | mutagenesis |
| **BA0672KO2** | 5’-acaggtaccctttagatgttggaacttgtccattca-3’ | mutagenesis |
| **BA1295KO1** | 5’-acaggtaccccagtctgtatatgctgaaacgc-3’ | mutagenesis |
| **BA1295KO2** | 5’-acaggtacctaccatttagaccacctctaactg-3’ | mutagenesis |
| **BA4109KO1** | 5’-acaggtaccttagtaaaaggtagaaaattagcg-3’ | mutagenesis |
| **BA4109KO2** | 5’-acaggtaccctcaaacttatggccatctggatct-3’ | mutagenesis |
| **Bcp25KO1** | 5’-cattagtcgaacacaacgctcgacttg-3’ | mutagenesis |
| **Bcp25KO2** | 5’-gaagttctgttcttcaagtagttgcc-3’ | mutagenesis |
| **Wip39KO1** | 5’-gataaagaacggactgctgtagcttgg-3’ | mutagenesis |
| **Wip39KO2** | 5’-agttgtattccagataggctatgtcc-3’ | mutagenesis |
| **PlyG BD1** | 5’-acagatatcattcagaaggttaagaatgg-3’ | GFP-PlyG^BD^ fusion |
| **PlyG BD2** | 5’-cccaagcttttatttaacttcataccacc-3’ | GFP-PlyG^BD^ fusion |
| **bcp25-1 up** | 5’-gatacttcaaaaggactacagttttcc-3’ | RT-PCR |
| **bcp25-2 down** | 5’-cgtactttaatgtaagtacggattg-3’ | RT-PCR |
| **bcp25-3 mid** | 5’-cggtacatacgcaaataagatatctc-3’ | RT-PCR |
| **bcp25-4 up** | 5’-cgatggattgacgcagagattggcaac-3’ | RT-PCR |
| **bcp25-5 down** | 5’-cgtaaaatgctaaacctgagaaccagtc-3’ | RT-PCR |
| **bcp26-1 up** | 5’-tacgcattcagtacgtacgcttatc-3’ | RT-PCR |
| **bcp26-2 down** | 5’-cctttagaatgtgctcacagaac-3’ | RT-PCR |
| **bcp26-3 mid** | 5’-ccttcttgaaacatgtcttcgaactcg-3’ | RT-PCR |
| **bcp26-4 up** | 5’-gatatcgactcaggttacgcattcagtac-3’ | RT-PCR |
| **bcp26-5 down** | 5’-caccacctccttcaatgttgcgg-3’ | RT-PCR |
| **wip38-1 up** | 5’-atggctaataataaaaacggg-3’ | RT-PCR |
| **wip38-2 down** | 5’-ccaaactgagttaaatcttcaatatc-3’ | RT-PCR |
| **wip38-3 up** | 5’-gcactttcctaagtttgataaaggagag-3’ | RT-PCR |
| **wip38-4 down** | 5’-ccgataattttaggaacagcatatg-3 | RT-PCR |
| **wip39-1 up** | 5’-cctgaattcggatgtgcattttcaac-3’ | RT-PCR |
| **wip39-2 down** | 5’-cagctttaaattctcttattac-3’ | RT-PCR |
| **wip39-3 up** | 5’-ccctaaagtatcaaccaagtac-3’ | RT-PCR |
| **wip39-4 down** | 5’-gagcagctctttgattaatctttgc-3’ | RT-PCR |
| **sap-1 up** | 5’-gctggcgttattaaaggtacagg-3’ | RT-PCR |
| **sap-2 down** | 5’-atcgctttgttagttactttgat-3’ | RT-PCR |
| **sigF-1 up** | 5’-accgcggatacgaaccagacg-3’ | RT-PCR |
| **sigF-2 down** | 5’-gatcaaattgcagatcaatctga-3’ | RT-PCR |
