# Supplementary figures and images for: The Secret Life of the Anthrax Agent Bacillus anthracis: Bacteriophage-Mediated Ecological Adaptations
Source: PLoS One. 2009 Aug 12;4(8):e6532. doi: 10.1371/journal.pone.0006532 (PMC2716549; doi:10.1371/journal.pone.0006532)

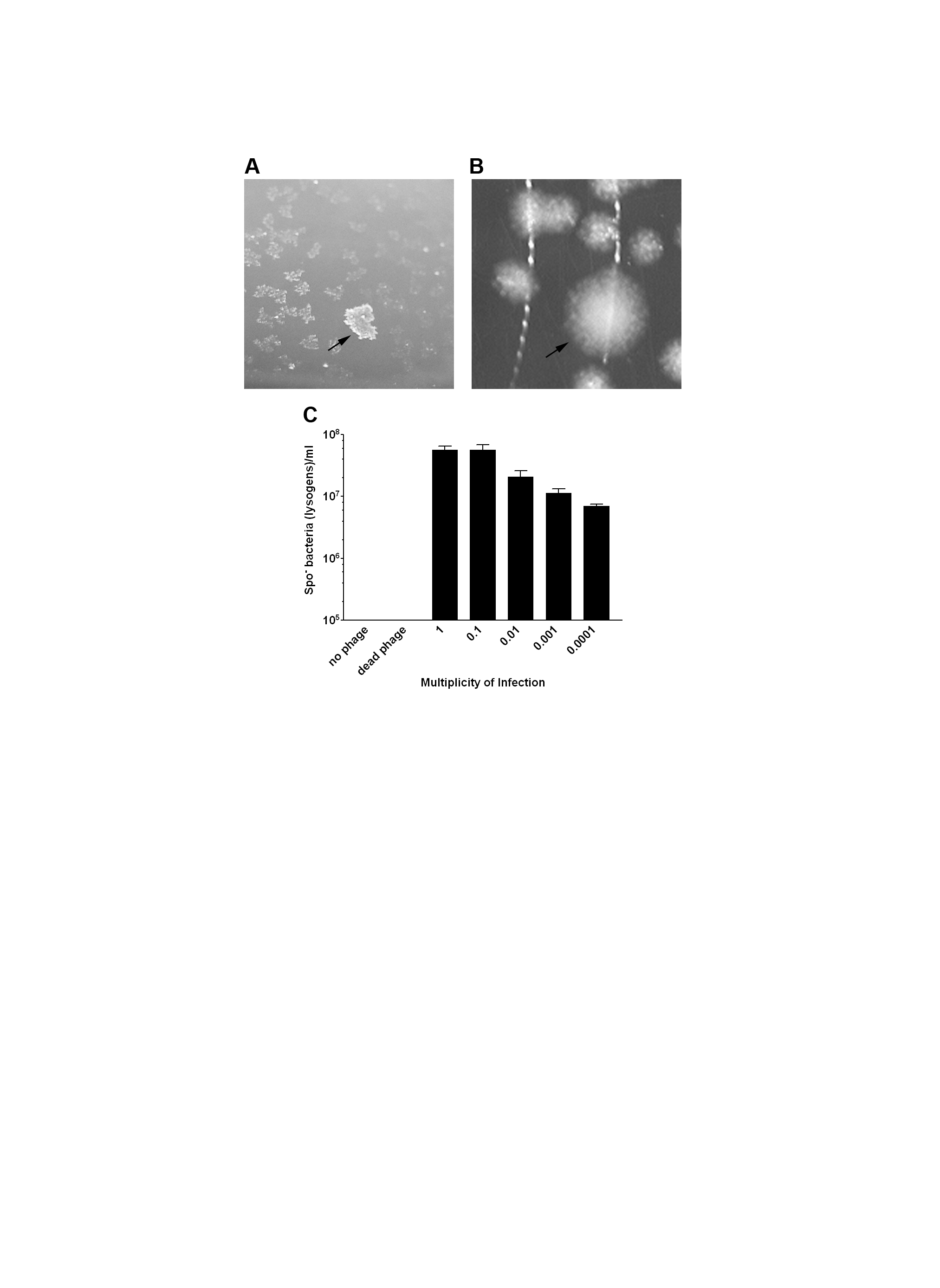

Supplement: Figure S1 — The appearance of asporogenous (Spo−) B. anthracis lysogens. (A) A Spo− Wip4 lysogen (indicated by arrow) appearing in field of Spo+ non-lysogens. Here, B. anthracis strain ΔSterne was infected with Wip4 (MOI of 0.01) for 16 hours in BHI liquid culture and plated for 24 hours on soil-extract agar. The indicated colony is ∼1–2 mm in diameter. (B) A Spo− Frp1 lysogen (indicated by arrow) in field of Spo+ non-lysogens. Indicated colony is 4 mm in diameter. (C) The appearance of Spo− derivatives of ΔSterne in Wip4-infected cultures. Here, mid-log phase liquid BHI cultures were infected with a range of phages concentrations (MOIs) for 3 hours, washed and plated for 16 hours on BHI. Resulting colonies were screened by PCR with phage-specific primers to identify lysogens. All lysogens corresponded to Spo− colonies. Numbers are mean averages (n = 10) of Spo− lysogens appearing in each condition and the error bars are standard deviations. (0.99 MB TIF) [file pone.0006532.s008.tif]

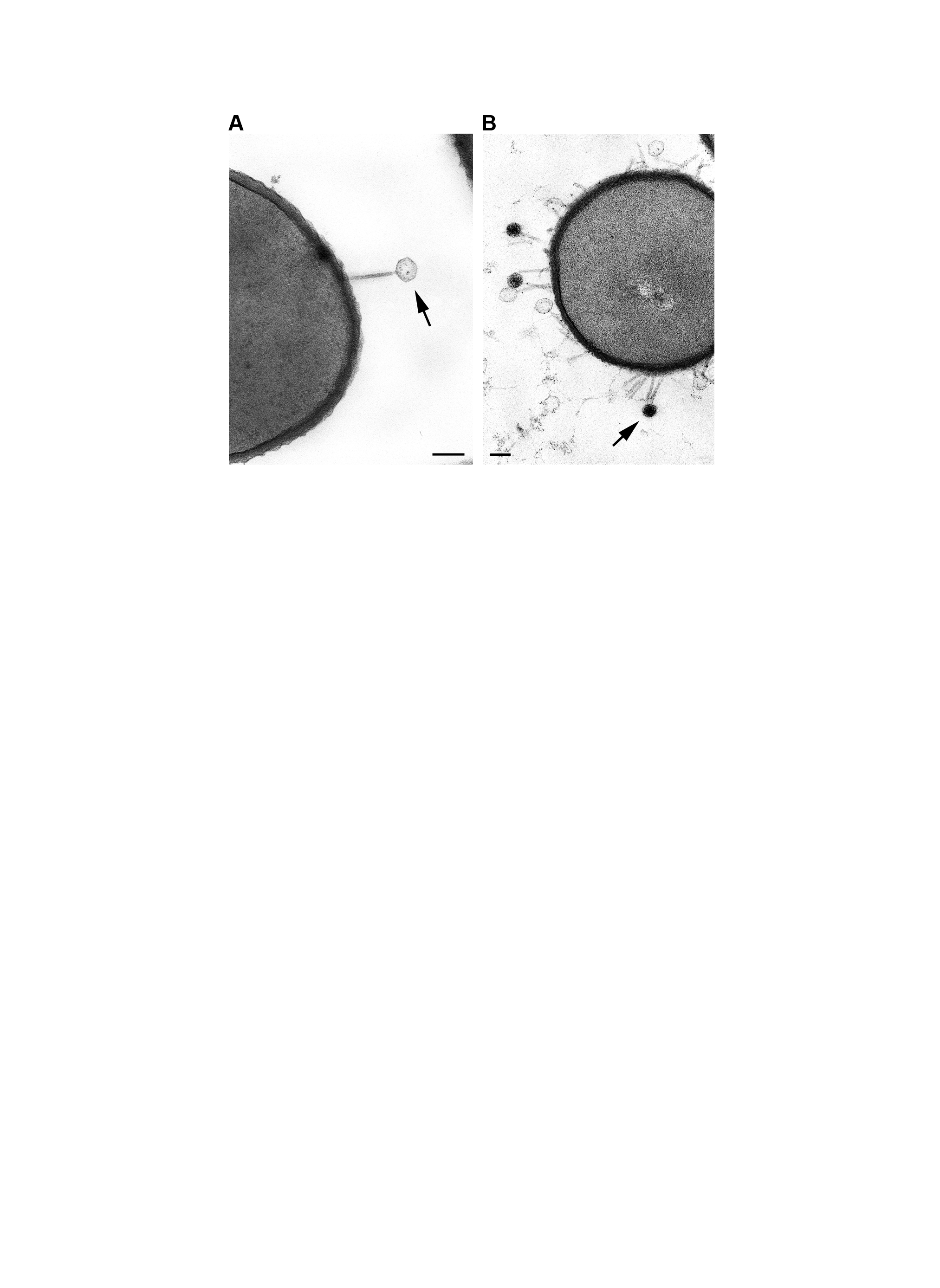

Supplement: Figure S2 — Bcp1 adsorbs to B. anthracis ΔSterne. Bacteria were infected with Bcp1 at an MOI of 1 (A) or 50 (B) for 15 minutes at 37°C, washed twice with PBS, fixed, and analyzed by thin-section electron microscopy. Scale bars are 50 nm. Arrows indicate phage heads that are either free (A) or full (B) of the Bcp1 genome. The absence of DNA in the phage head suggests that the genome translocated into ΔSterne. (1.77 MB TIF) [file pone.0006532.s009.tif]

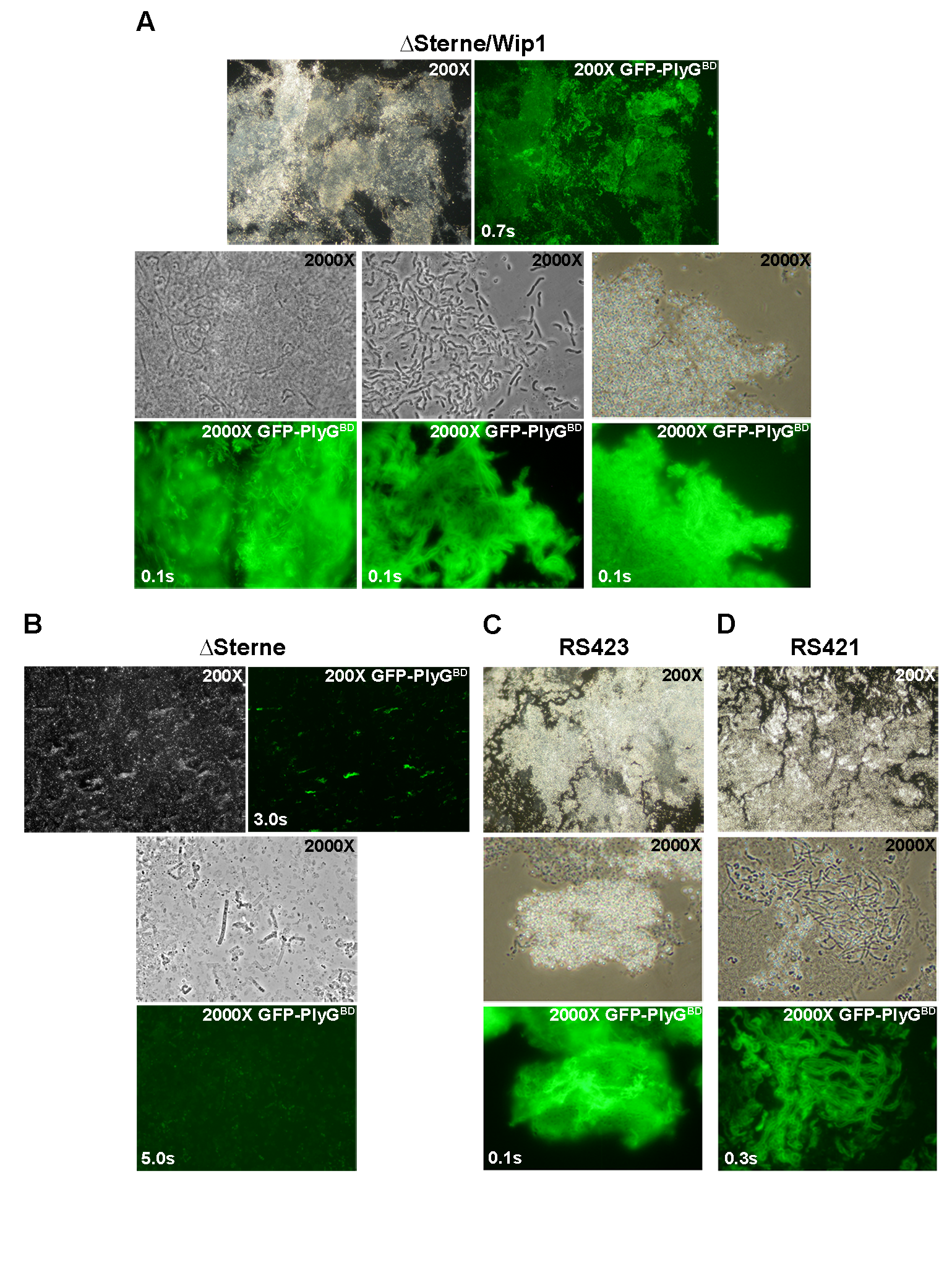

Supplement: Figure S3 — Biofilms formed by B. anthracis and environmental B. cereus strains. Either settled material (for ΔSterne) or biofilms (for ΔSterne/Wip1, RS423, and RS421) formed at the liquid-air interface of 3 month-old BHI cultures grown without aeration at 24°C were recovered, labeled with GFP-PlyGBD, and examined by phase-constrast and fluorescence microscopy at 200X and 2000X magnifications. Exposure times are indicated for fluorescence images. (A) The biofilms of ΔSterne/Wip1 consist of a matrix enriched with the B. anthracis exopolysaccharide (the binding target of GFP-PlyGBD). Three distinct regions are observed in 2000X images, including spore/vegetative mixtures, vegetative-enriched, and spore-enriched zones from left to right. (B) Settled material in 3 month ΔSterne cultures consists predominantly of cellular debris that does not bind well to GFP-PlyGBD. ΔSterne alone does not form biolfims, thus only the settled material was analyzed. (C) Biofilms formed by RS423, a B. cereus s.l. strain from the worm gut, are in a GFP-PlyGBD-labeled matrix. (D) Biofilms formed by RS421, a B. cereus s.l. strain from the worm gut, are in a GFP-PlyGBD-labeled matrix. (8.21 MB TIF) [file pone.0006532.s010.tif]

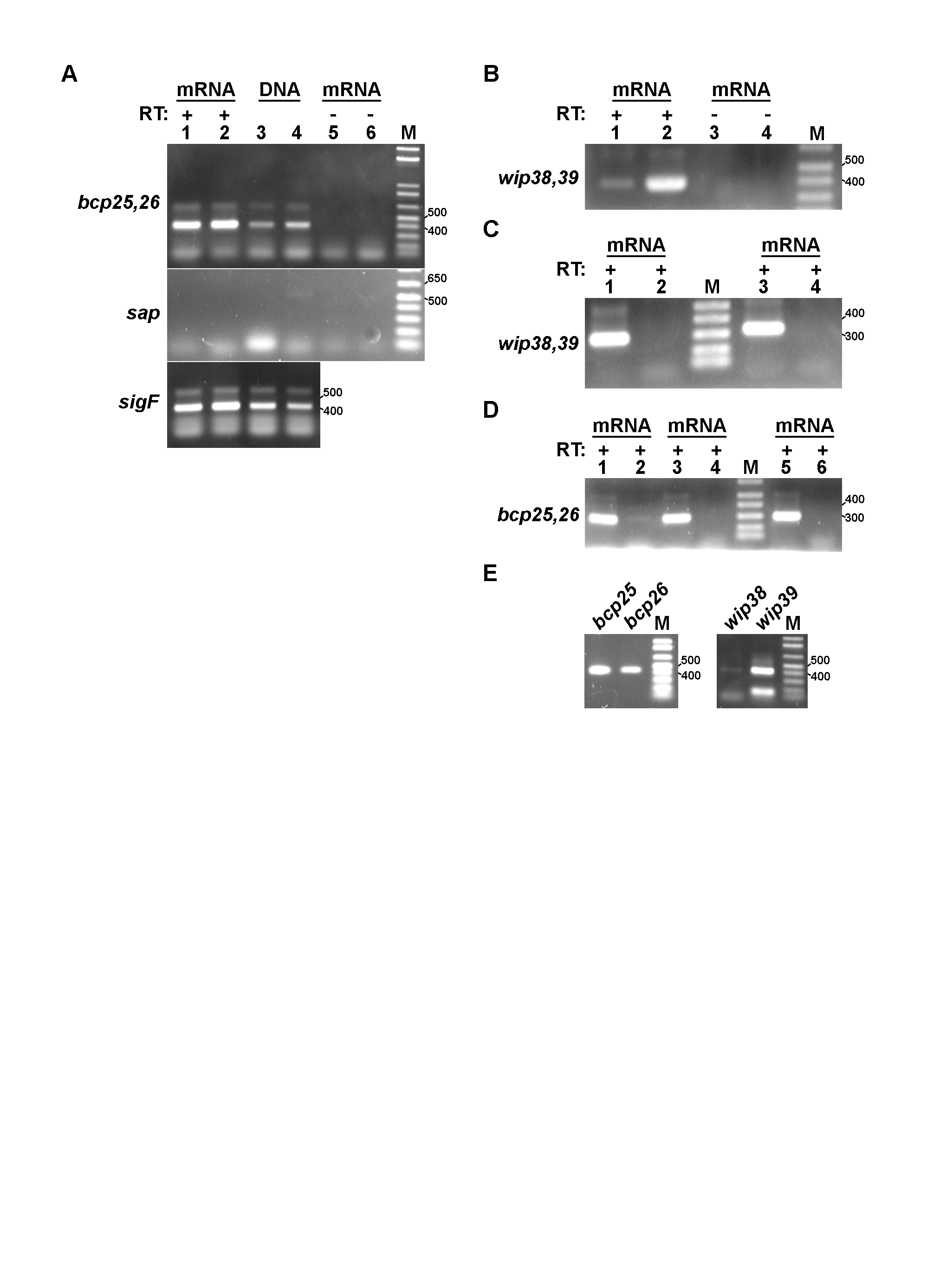

Supplement: Figure S5 — RT-PCR analysis of bcp25,26 and wip38,39 mRNA. We extracted mRNA from ΔSterne derivatives grown for 3 hours at 37°C in LD (A–D) or BHI (E) medium. The mRNA samples incubated either with (+) or without (−) reverse transcriptase (RT). Resulting cDNA was analyzed with primers listed to the left of each panel (sequences are in Table S7). Lane M is the 1 Kb Plus DNA ladder (New England Biolabs). Sizes in base pairs are at the right of each gel. (A) The ΔSterne/Bcp1 lyosgen. Amplifications were performed with primers bcp25-1,2 (lanes 1, 3 and 5) or bcp26-1,2 (lanes 2, 4, and 6) using either genomic DNA (lanes 3 and 4) or RT-treated (lanes 1 and 2) and untreated (lanes 5 and 6) mRNA. As controls, primers for sap (BA0885, a locus expressed during vegetative growth) and sigF (BA4294, a locus expressed only during sporulation) were used. (B) The ΔSterne/Wip4 lyosgen. Amplifications were performed with primers wip38-1,2 (lanes 1 and 3) or wip39-1,2 (lanes 2 and 4) using RT-treated (lanes 1 and 2) and untreated (lanes 5 and 6) mRNA. The wip38-1,2 primers span the intergenic region of wip38 and wip39. (C) Analysis of ΔSterne/pASD2::P-wip38,39 (Lanes 1 and 3) or ΔSterne/pASD2:: wip38,39 PROMOTERLESS (lanes 2 and 4). The RT-treated samples were amplified with primers wip38-3,4 (lanes 1 and 2) or wip39-3,4 (lanes 3 and 4). The wip38-3,4 primers span the intergenic region of wip38 and wip39. (D) Analysis of ΔSterne/pASD2::P-bcp25,26 (Lanes 1, 3, and 5) or ΔSterne/pASD2::bcp25,26 PROMOTERLESS (lanes 2, 4, 6). Amplifications were performed on RT-treated samples using primers bcp25-4,5 (lanes 1 and 2), bcp26-4,5 (lanes 3 and 4), or bcp25-3,bcp26-3 which span the bcp25-bcp26 intergenic region (lanes 5 and 6). (E) Analysis ΔSterne lysogens during vegetative growth in BHI. Amplifications were performed on RT-treated samples from ΔSterne/Bcp1 (with bcp25-1,2 and bcp26-1,2 primers) and ΔSterne/Wip4 (with wip38-1,2 and wip39-1,2 primers). (4.50 MB TIF) [file pone.0006532.s012.tif]

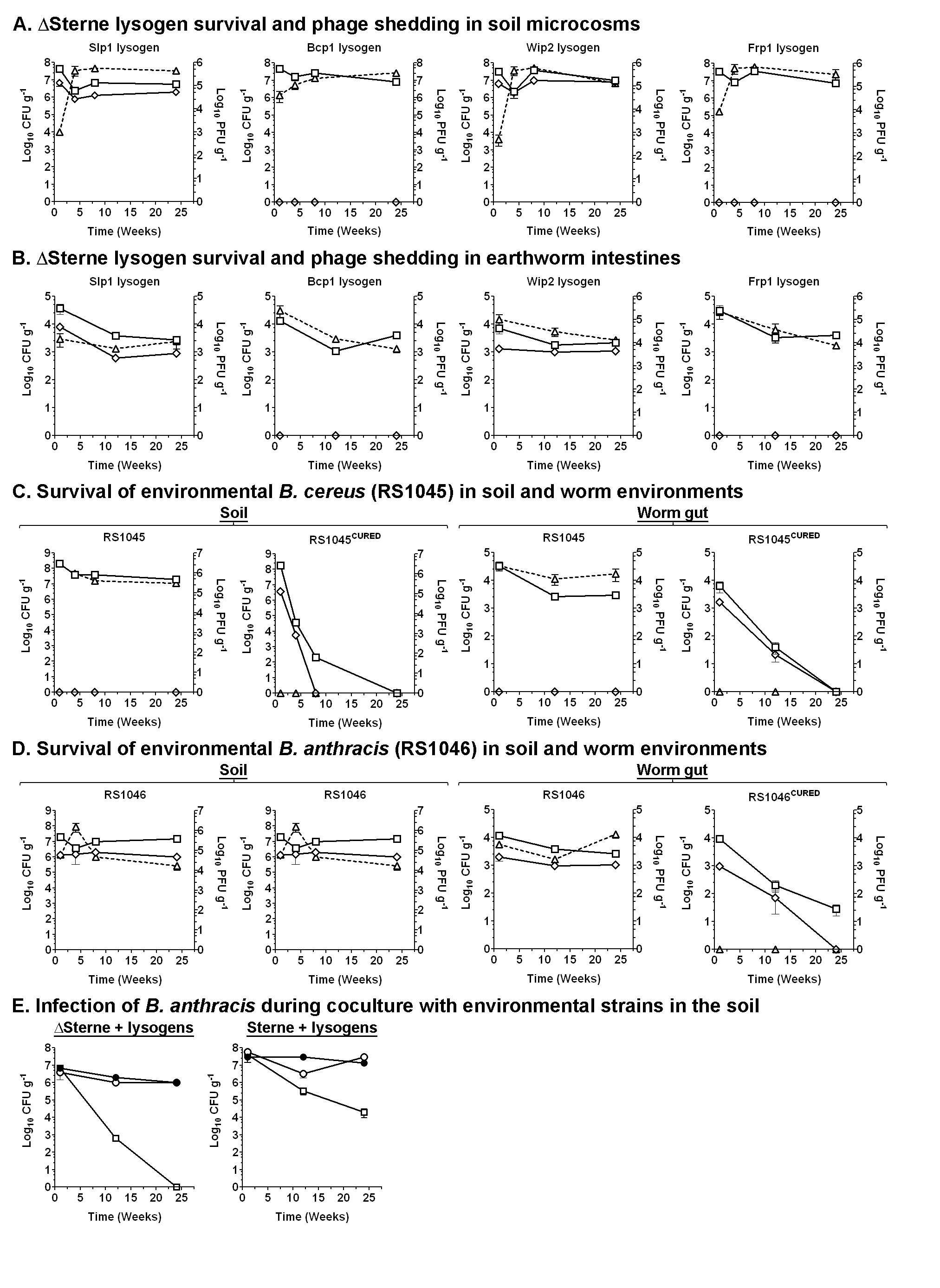

Supplement: Figure S6 — Expanded analysis of survival in the soil and earthworm. Survival at indicated times after inoculation (solid lines) is shown as CFUs per gram of recovered soil or worm guts. Similarly, shedding of free phages (dashed lines) is shown as PFUs extracted per gram of soil or worm guts. Data is shown for B. anthracis viability (vegetative cells and spores; squares), spores alone (diamonds), and free phages (triangles). Values are reported as mean averages (n = 5) and error bars are standard deviations. (A) Soil survival for ΔSterne/pASD2 lysogens. (B) Earthworm gut survival for ΔSterne/pASD2 lysogens. (C) Survival of environmental B.cereus strain RS1045 and its phage-cured derivative (RS1045CURED) in the soil and earthworm gut. (D) Survival of environmental B. anthracis strain RS1046 and its phage-cured derivative (RS1046CURED) in the soil and earthworm gut. (E) Infection of B. anthracis during co-culture with lysogens in soil microcosms. Strains ΔSterne/pASD2 and Sterne/pASD2 were either inoculated alone or with B. cereus RS1045 or B. anthracis RS1046. At the indicated time points, ΔSterne/pASD2 and Sterne/pASD2 were selectively recovered and scored by PCR for infection with either Wip4 (the phage shed by RS1045) or Wip5 (the phage shed by RS1046). Survival of ΔSterne/pASD2 and Sterne/pASD2 inoculated alone (squares) and their derivatives that have become stably infected with Wip4 (closed circles) or Wip5 (open circles) are shown. (2.16 MB TIF) [file pone.0006532.s013.tif]

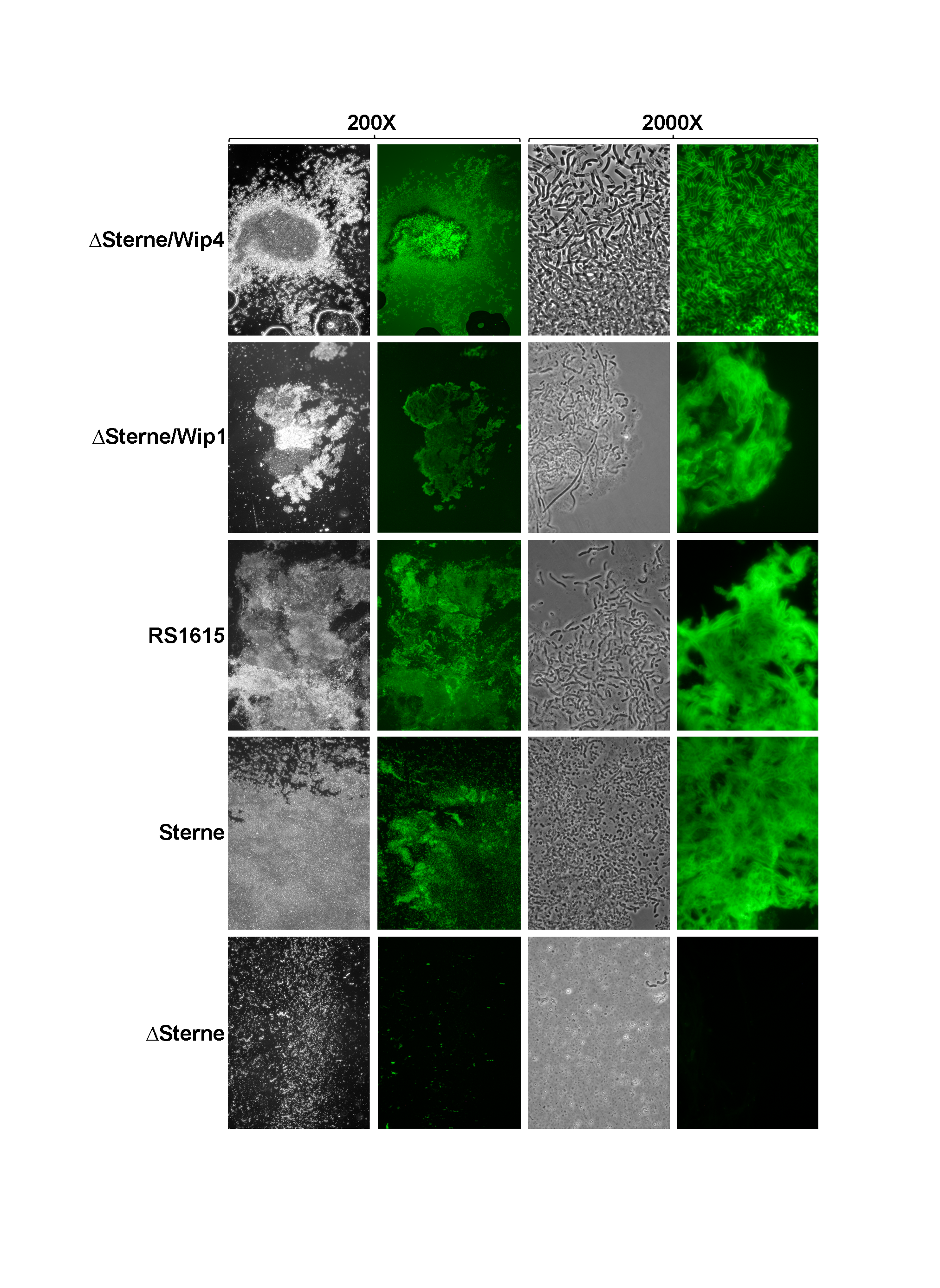

Supplement: Figure S7 — Microscopic analysis of B. anthracis strains recovered from soil microcosms. Culture aliquots of indicated strains were removed at 3 months, labeled with GFP-PlyGBD, and analyzed. Phase-contrast and corresponding fluorescence images are shown at 200X and 2000X magnification. The exposure time for each image was 0.3 seconds. (9.51 MB TIF) [file pone.0006532.s014.tif]

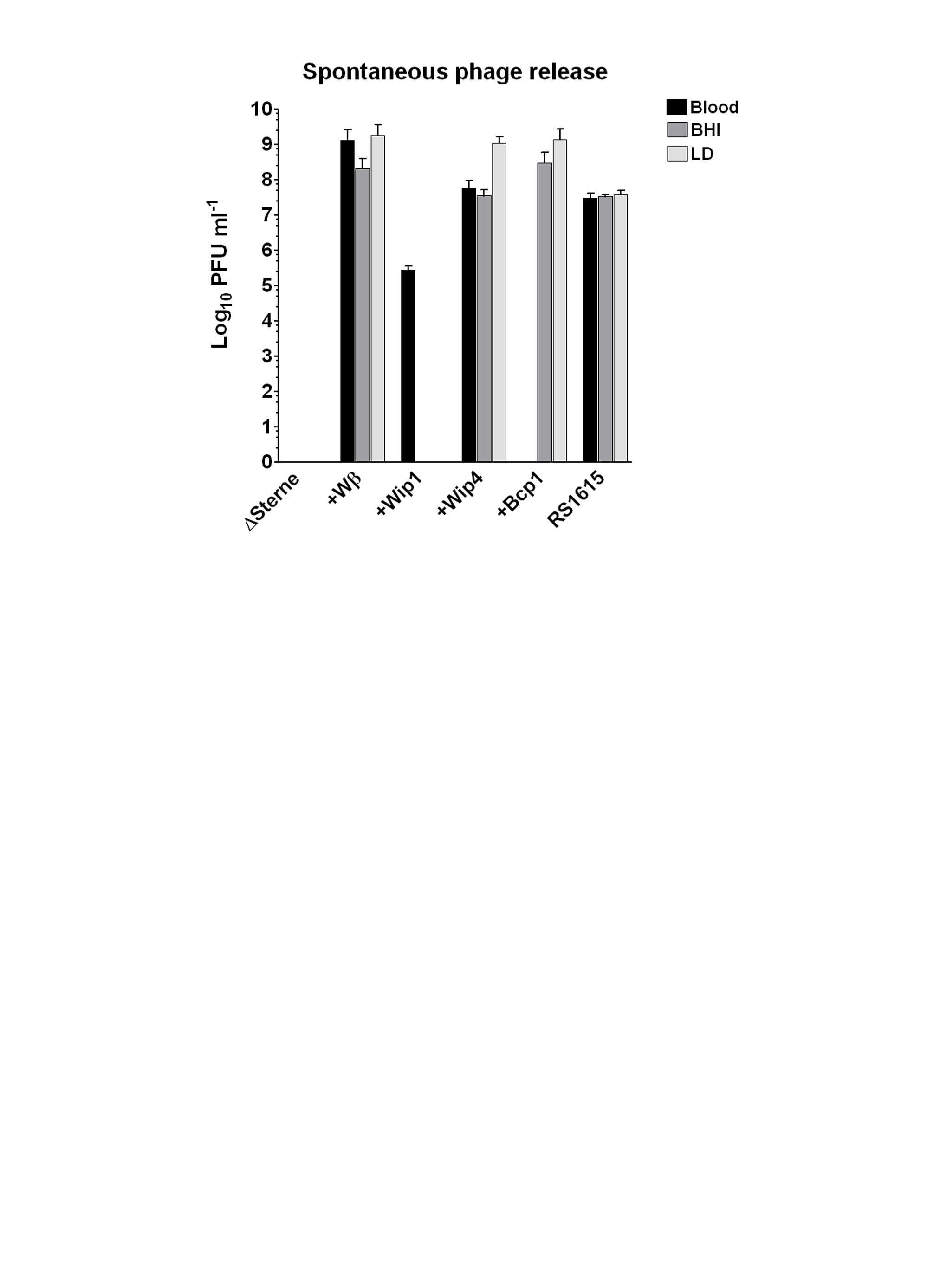

Supplement: Figure S8 — The shedding of bacteriophage by B. anthracis and its lysogens. B. anthracis strain ΔSterne, its indicated lysogens, and the environmental B. anthracis strain RS1615 were examined. Numbers are mean averages (n = 5) of PFUs shed into the media during the culture of each stain and the error bars are standard deviations. (1.09 MB TIF) [file pone.0006532.s015.tif]
