# Supplementary material for: The Secret Life of the Anthrax Agent Bacillus anthracis: Bacteriophage-Mediated Ecological Adaptations
Source: PLoS One. 2009 Aug 12;4(8):e6532. doi: 10.1371/journal.pone.0006532 (PMC2716549; doi:10.1371/journal.pone.0006532)
Supplement: Figure S4 — Amino acid sequence alignment of known and putative bacterial sigma factors. Identical residues are highlighted by black backgrounds. Conserved amino acid changes are highlighted by gray backgrounds. SigF is the B. anthracis sigma factor, σF, encoded by BA4294. The alignment was generated by ClustalW and displayed using the BOXSHADE program. (0.03 MB DOC) [file pone.0006532.s011.doc]

Wip39 1 -----------MTREKGQAKEIVDVRGMSDDEFMEKYERLVHHCVWKRYAKKRPAIEHDTNLDIEDLTQFGMIGLIKARD
Bcp25 1 MNKKRAGSTYTFAKKVLTKEETYELIERSQAGEEEATESLVEHNARLVTYVVRRKN-NPY-HEYDDLFQLGMIGLITAIA
Bcp26 1 ------MILWKVRGIELTREETIELVKEARAGSEEAKEKIILGYKGMVYTLAKRFSRSKQ-HEFEDMFQEGLTILLEVVD
SigF 1 ---MDIEVKNEKKKPQLKDHELKALIQKSQDGDQQARDTIVQSNMRLVWSVVQRFL-NRG-YEPDDLFQIGCIGLLKSVD


Wip39 70 NFNPEFGCAFSTYAVPKIIGEIGRAIRD---NQKVKVQRSVYSVKGKILNQQLSDKGPEEIADILNEPVTLVKMALKYQP
Bcp25 79 KFDTSKGLQFSTYAVRWIDAEIGNYLKN--RTSILKVPREIGAIVNKILAVKLKNEEPAIIMEKLKLDASQLDNVTIALE
Bcp26 74 KFDIDSGYAFSTYAYPFVFGKMNNVRKR---YNPIKISAHITDIISRIRKYKLTDRSEKEIYEFLNKEYELKWVRAALEY
SigF 76 KFDLSFDVKFSTYAVPMIIGEIQRFLRDDGSVKVSRSLKETGNKIRKMRDELSKEFGRAPTINEVAEALELTPEEVVLAQ


Wip39 147 S-TDSLNKVVYASGSNEDVTLEKMLEDTKVEDIEETTINRAVIREFKAALAP-KEYIVLDMHLQNMTQQNIANQMGYSQV
Bcp25 157 IIHNEVISLDKQTGEEKDD----SLSSIVGQDVNQDWFSGLAFYDIIRFLDDKEQSVLTLKYVHDMSSNKIATLFGTYAN
Bcp26 151 MRRGKVLSLEKTFAEDEESDWAATLKEVVSKDANGDWELMMDIKGCVPSLTSYEQYAFCEHILKDRMQSDIAEELGVKPQ
SigF 156 EASRAPSSIHETVYENDGD--PITILDQIADQSETKWFDKIALKEAIRELDERERLIVYLRYYKDQTQSEVAERIGISQV


Wip39 225 QISRILAKINQRAAQFGKEGGLQD
Bcp25 233 KISRLEKVALDKLRERYTYEELIN
Bcp26 231 TVSKHVNKACRKIKLELGGV----
SigF 234 QVSRLEKKILKQMKDRIDE-----
